# Unusual illudin-type sesquiterpenoids from cultures of *Agrocybe salicacola*

**DOI:** 10.1007/s13659-011-0018-4

**Published:** 2011-10-30

**Authors:** Liang-Yan Liu, Ling Zhang, Tao Feng, Zheng-Hui Li, Ze-Jun Dong, Xing-Yao Li, Jia Su, Yan Li, Ji-Kai Liu

**Affiliations:** 1 State Key Laboratory of Phytochemistry and Plant Resources in West China, Kunming Institute of Botany, Chinese Academy of Sciences, Kunming, 650201 China; 2 Graduate University of Chinese Academy of Sciences, Beijing, 100039 China

**Keywords:** *Agrocybe salicacola*, sesquiterpenoid, agrocybin, stereoconfiguration, X-ray

## Abstract

Seven new illudin-type sesquiterpenoids, agrocybins A–G (**1**–**7**), along with three known analogues (**8**–**10**), have been isolated from the culture broth of the fungus *Agrocybe salicacola*. Their structures were elucidated on the basis of extensive spectroscopic data analysis and comparison with data reported in the literature. The relative stereoconfigurations of **1** and **6** were elucidated by the X-ray crystallographic diffraction analysis. Compound **1** was highly cyclized containing seven chiral carbons which arranged compactly in six rings.

**Electronic Supplementary Material:**

Supplementary material is available for this article at 10.1007/s13659-011-0018-4 and is accessible for authorized users.

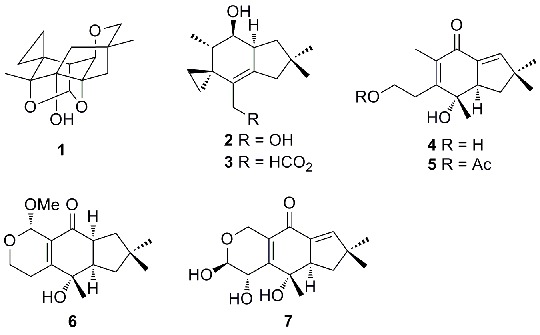

## Introduction

The genus *Agrocybe* contains 174 species and varietas, and has been reported to produce rich bioactive metabolites such as ceramides, indole alkaloids, peptides, lectin, and polysaccharides, with inhibitory activity against COX-1 and COX-2, free radical scavenging ability, antifungal activity, mitogenic effect, and hypoglycemic function.[1–5] *Agrocybe salicacola* (Chinese name, Yang-Liu-Tian-Tou-Gu) is a delicate edible mushroom endemic to Yunnan province, People's Republic of China, which grows at trunks of poplars and willows from September to October.[6–7] Our previous investigation on the cultures of this fungus revealed a novel illudane-illudane bis-sesquiterpene (agrocybone) and an illudin derivative.[8,9] Surveys on the literature, we found that illudin-type sesquiterpenoids may play an important role on the antitumor property.[10] For instance, irofulven, a derivative of natural occuring illudin S from the fruiting bodies or culture broth of several species in basidiomycetes,[11] has been admitted to clinical trails for the treatment of cancers.[12–14] In order to search for more illudin-type sesquiterpenoids, especially ones with high cytotoxicity, we enlarged the scale of fermentation of *A. salicacola*, and isolated seven new illudin-type sesquiterpenoids, named as agrocybins A–G (**1**–**7**), together with three known analogues. The new structures were determined on the basis of extensive spectroscopic analysis and the X-ray crystallographic diffraction analysis, while the known compounds were identified as (2*R*)-2,3-dihydro-7-hydroxy-2-(hydroxymethyl)-2,4,6-trimethyl-1H-indene-5-ethanol (**8**),[15] illudin T (**9**),[9] and agrocybone (**10**)[8] by comparison with data reported in the literature. Compounds **1**–**10** were tested for their cytotoxic activity. All these compounds showed no cytotoxic activity against human tumor cell lines HL-60, SMMC-7712, A-549, MCF-7, and SW480 *in vitro*.

*To whom correspondence should be addressed. E-mail: jkliu@mail.kib.ac.cn

Springer

**Table 1.** $^{13}C$ **NMR spectroscopic data for compounds 1–7.**

| Position | **1**[a] | **2**[b] | **3**[c] | **4**[d] | **5**[d] | **6**[c] | **7**[b] |
|---|---|---|---|---|---|---|---|
| 1 | 78.1, CH | 75.8, CH | 74.4, CH | 186.3, C | 186.1, C | 200.2, C | 187.0, C |
| 2 | 49.9, CH | 46.8, CH | 46.3, CH | 132.9, C | 134.5, C | 130.4, C | 132.2, C |
| 3 | 27.6, C | 26.6, C | 26.5, C | 163.4, C | 160.1, C | 157.3, C | 158.6, C |
| 4 | 88.6, C | 129.2, C | 124.2, C | 74.2, C | 75.4, C | 70.3, C | 75.3, C |
| 5 | 77.6, C | 142.3, C | 145.7, C | 54.2, CH | 54.4, CH | 52.7, CH | 55.7, CH |
| 6 | 38.6, $CH_2$ | 44.7, $CH_2$ | 44.8, $CH_2$ | 39.7, $CH_2$ | 39.6, $CH_2$ | 44.7, $CH_2$ | 40.7, $CH_2$ |
| 7 | 37.8, C | 38.2, C | 37.7, C | 45.1, C | 45.2, C | 38.0, C | 46.2, C |
| 8 | 41.6, $CH_2$ | 46.6, $CH_2$ | 46.5, $CH_2$ | 147.8, CH | 148.0, CH | 42.3, $CH_2$ | 149.8, CH |
| 9 | 84.7, C | 44.7, CH | 44.8, CH | 137.1, C | 136.8, C | 49.1, CH | 138.4, C |
| 10 | 103.9, CH | 11.4, $CH_3$ | 11.4, $CH_3$ | 11.5, $CH_3$ | 12.0, $CH_3$ | 94.6, CH | 57.8, $CH_2$ |
| 11 | 5.3, $CH_2$ | 14.9, $CH_2$ | 14.3, $CH_2$ | 30.5, $CH_2$ | 28.9, $CH_2$ | 25.5, $CH_2$ | 64.4, CH |
| 12 | 6.4, $CH_2$ | 10.0, $CH_2$ | 9.5, $CH_2$ | 61.5, $CH_2$ | 63.5, $CH_2$ | 57.3, $CH_2$ | 94.3, CH |
| 13 | 11.6, $CH_3$ | 58.0, $CH_2$ | 59.5, $CH_2$ | 22.7, $CH_3$ | 22.3, $CH_3$ | 24.9, $CH_3$ | 22.2, $CH_3$ |
| 14 | 78.5, $CH_2$ | 30.2, $CH_3$ | 30.3, $CH_3$ | 27.7, $CH_3$ | 21.8, $CH_3$ | 31.8, $CH_3$ | 27.8, $CH_3$ |
| 15 | 21.6, $CH_3$ | 31.1, $CH_3$ | 30.8, $CH_3$ | 28.5, $CH_3$ | 28.6, $CH_3$ | 31.9, $CH_3$ | 28.7, $CH_3$ |
| H*C*O | | | 161.9, CH | | | | |
| *C*$H_3$CO | | | | | 21.0, $CH_3$ | | |
| $CH_3$*C*O | | | | | 171.5, C | | |
| O*C*$H_3$ | | | | | | 55.9, $CH_3$ | |

[a]spectra were measured in $CDCl_3$ at 125 MHz; [b]spectra were measured in acetone-$d_6$ at 125 MHz; [c]spectra were measured in methanol-$d_4$ at 100 MHz; [d] spectra were measured in $CDCl_3$ at 100 MHz.

## Results and Discussion

Compound **1** was obtained as colorless needles. The IR spectrum displayed an absorption band at 3425 $cm^{-1}$ due to the hydroxy group. The positive HRESIMS showed a pseudo molecular ion at *m/z* 287.1251 ($[M + Na]^+$), corresponding to the molecular formula $C_{15}H_{20}O_4$ that required six degrees of unsaturation. Inspection of the $^1H$ and $^{13}C$ NMR (DEPT) and HSQC spectra revealed the existence of two methyl groups, five methylene groups (including an oxymethylene), three methenyl units (including an acetal unit) and five quaternary carbons (two of them are oxygen-bearing ones) (Tables 1 and 2). These data suggested that **1** might be a six-ring sesquiterpenoid.

In the $^{13}C$ NMR data, three characteristic signals at $\delta_C$ 27.6 (s, C-3), 5.3 (t, C-11), and 6.4 (t, C-12) revealed the existence of a three-membered carbon skeleton (ring A).[8,9] These information, along with the characteristic quaternary carbon resonance at $\delta_C$ 37.8 (s, C-7) and four methyl groups (including two oxidated ones) led to the hypothesis that compound **1** might be an illudin-type sesquiterpenoid possessing a 3/6/5 carbon skeleton (rings A–C as shown in Figure 1) as those reported previously from the same resource.[8,9] Detailed analysis of $^1H$-$^1H$ COSY and HMBC spectra supported the above hypothesis (Figure 1). Further, the proton of an acetal signal at $\delta_H$ 5.68 (1H, d, $J$ = 2.9 Hz, H-10) showed key HMBC correlations to two oxyquaternary carbon signals at $\delta_C$ 88.6 (s, C-4) and 84.7 (s, C-9), which established two five-membered rings D and E (Figure 1). In addition, the HMBC correlation of the proton of an oxymethine at $\delta_H$ 3.64 (1H, d, $J$ = 6.4 Hz, H-1) to the oxymethylene carbon at $\delta_C$ 78.5 (t, C-14) established ring F as shown in Figure 1. To the best of our knowledge, compound **1** was the first highly cyclized illudin-type sesquiterpenoid. Construction of a molecular model of **1** suggested that a six-ring system made the backbone compact. An X-ray diffraction analysis of **1** not only confirmed the structure as elucidated above but also established the relative configuration as shown in Figure 1. Thus, compound **1** was established and named as agrocybin A.

**Figure 1.** Key 2D NMR correlations and the X-ray structure of **1**.

Compound **2** was isolated as a colorless oil. Its molecular formula was determined to be $C_{15}H_{24}O_2$ by the pseudo molecular ion at *m/z* 259.1676 ($[M + Na]^+$) in the HRESIMS. The IR spectrum showed absorption at 3439 $cm^{-1}$ due to the hydroxy groups. $^{13}C$ NMR data analysis suggested that **2** was also an illudin-type sesquiterpenoid as deduced from the characteristic signals at $\delta_C$ 26.6 (s, C-3), 14.9 (t, C-11), and 10.0 (t, C-12) for the three-membered carbon ring and the quaternary resonance at $\delta_C$ 38.2 (s, C-7). 2D NMR data analysis indicated that compound **2** was structurally resembled illudane,[16] except for the terminal double bond between C-2 and C-10 becoming saturated in **2**, as supported by the HMBC correlation of $\delta_H$ 0.97 (3H, d, $J$ = 6.8 Hz, H-10) with $\delta_C$ 46.8 (d, C-2), 75.8 (d, C-1), and 26.6 (s, C-3). The relative configuration was elucidated by the ROESY experiment. The ROESY correlations of H-1/H-10 and H-10/H-9 suggested that H-9 and Me-10 were on the

**Table 2.** $^{1}H$ **NMR spectroscopic data for compounds 1–3.**

| Pos. | **1**[a] | **2**[b] | **3**[c] |
|---|---|---|---|
| 1 | 3.64, d (6.4) | 3.68, dd (9.7, 3.9) | 3.71, m |
| 2 | 2.37, m | 1.15, m | 1.16, m |
| 6a | 1.85, d (12.7) | 2.27, m | 2.27, d (17.0) |
| 6b | 1.39, d (12.7) | | 2.21, d (17.0) |
| 8a | 2.43, d (9.8) | 1.89, dd (11.8, 7.1) | 1.91, dd (11.8, 7.4) |
| 8b | 1.34, d (9.8) | 1.17, dd (11.8, 11.5) | 1.18, m |
| 9 | | 2.70, m | 2.72, m |
| 10 | 5.68, d (2.9) | 0.97, d (6.8) | 0.95, d (6.9) |
| 11a | 0.68, m | 0.69, m | 0.68, m |
| 11b | 0.07, m | 0.40, m | 0.44, m |
| 12a | 1.28, m | 0.98, m | 0.86, m |
| 12b | 0.76, m | 0.59, m | 0.59, m |
| 13a | 0.95, s | 3.78, d (11.5) | 4.40, d (12.0) |
| 13b | | 3.51, d (11.5) | 4.12, d (12.0) |
| 14 | 3.43, s | 1.08, s | 1.06, s |
| 15 | 1.05, s | 1.08, s | 1.06, s |
| *H*CO | | | 8.09, s |

[a]spectra were measured in $CDCl_3$ at 500 MHz;
[b]spectra were measured in acetone-$d_6$ at 400 MHz;
[c]spectra were measured in methanol-$d_4$ at 500 MHz.

same side, while OH-1 on the opposite side. Therefore, compound **2** was established and named as agrocybin B.

$^{1}H$ and $^{13}C$ NMR data (Tables 1 and 2) of compound **3** were closely related to those of **2**, except for signals of one additional formyl group [$\delta_H$ 8.09 (1H, s, *H*CO); $\delta_C$ 161.9 (H*C*O)], which was supported by the IR absorption band at 1721 $cm^{-1}$. The formyl group was placed at OH-13 as supported by the HMBC correlation of $\delta_H$ 8.09 (1H, s, *H*CO) with $\delta_C$ 59.5 (t, C-13). Detailed analysis of 2D NMR data suggested that **3** is the C-13 monoformate of **2**. Therefore, compound **3** was elucidated as shown and named as agrocybin C.

Compound **4**, a colorless oil, was assigned the molecular formula of $C_{15}H_{22}O_3$ based on the positive HRESIMS at *m/z* 251.1649 ($[M + H]^+$), accounting for five degrees of unsaturation. Analysis of its $^{1}H$ and $^{13}C$ NMR data (Tables 1 and 3) revealed four methyls, three methylenes (one oxygenated), one methine, four olefinic carbons (one of which is protonated), two *sp*3 quaternary carbons (one oxygenated), and one carbonyl carbon. These data suggested compound **4** to be a dicyclic sesquiterpenoid related to illudalenol[17] except for one difference that the double bond between C-5 and C-9 migrated to between C-9 and C-8 in **4**, as supported by the HMBC cross-peaks from $\delta_H$ 6.52 (1H, d, $J$ = 2.5 Hz, H-8) to $\delta_C$ 45.1 (s, C-7) and from $\delta_H$ 3.38 (1H, ddd, $J$ = 8.2, 8.0, 2.5 Hz, H-5) to $\delta_C$ 137.1 (s, C-9). The ROESY correlations of H-5/H-6a and H-6b/H-13 suggested that H-5 and OH-4 were on the same side. Therefore, compound **4** was elucidated as shown and named as agrocybin D.

The elemental composition of compound **5** was established as $C_{17}H_{24}O_4$ (six degrees of unsaturation) by HRESIMS. Analysis of its 1D NMR spectroscopic data (Tables 1 and 3) revealed nearly identical structural features to those found in **4**, except that the oxygenated methylene protons of $CH_2$-12 at $\delta_H$ 4.00 and 3.80 in **4** were significantly downfield ($\delta_H$ 4.48 and 4.21, respectively) in **5**. In addition, NMR signals corresponding to an acetyl group ($\delta_H/\delta_C$ 2.04/21.0, 171.5) were observed, indicating that the C-12 oxygen of **5** was acylated, which was supported by the HMBC correlation from H-12 to the carboxylic carbon at $\delta_C$ 171.5. Consequently, agrocybin E (**5**) was assigned as the C-12 monoacetate of **4**.

Agrocybin F (**6**) was obtained as colorless needles. Its molecular formula was determined as $C_{16}H_{24}O_4$ on the basis of HRESIMS, implying five degrees of unsaturation. The $^{13}C$ NMR (DEPT) spectrum revealed the existence of 16 carbon signals, including four methyls (one methoxy group), four methylenes, three methines, and five quaternary carbons (Table 1). Besides one double bond and a carbonyl carbon, these data suggested compound **6** to be a tricyclic sesquiterpenoid. Preliminary analysis of 2D NMR data suggested that rings B and C of **6** were similar to those of **4** and **5** except that the double bond between C-8 and C-9 was saturated (Figure 2). In the HMBC spectrum, an acetal signal at $\delta_H$ 5.15 (1H, s, H-10) showed key correlations to $\delta_C$ 57.3 (t, C-12) and 130.4 (s, C-2), which established a new ring D as shown in Figure 2. In addition, the HMBC cross-peak from methoxy signal at $\delta_H$ 3.40 (3H, s, $OCH_3$) to the acetal carbon indicated the methoxy to be connected at C-10 (Figure 2). An X-ray diffraction assay finally established the structure of **6** and revealed its relative configuration as shown in Figure 2.

**Figure 2**. Key 2D NMR correlations and the X-ray structure of **6**.

Compound **7**, a colorless oil, had a molecular formula of $C_{15}H_{20}O_5$ (six degrees of unsaturation) on the basis of HRESIMS. Analysis of $^{13}C$ and $^{1}H$ NMR data (Tables 1 and 3) suggested that **7** possessed rings B and C identical to those of **4** and **5**. An oxygenated methylene [$\delta_H$ 4.41 (1H, d, $J$ = 16.5 Hz, H-10a) and 4.20 (1H, d, $J$ = 16.5 Hz, H-10b); $\delta_C$ 57.8] was assigned to C-10 on basis of HMBC correlations from H-10 to C-1, C-2, and C-3, while an oxygenated methine [$\delta_H$ 4.12 (1H, br. s, H-11); $\delta_C$ 64.4] was assigned to C-11 by the HMBC correlations from H-11 to C-2, C-3, and C-4, as well as the $^{1}H$-$^{1}H$ COSY correlation from H-11 to the acetal proton at $\delta_H$ 5.14 (1H, d, $J$ = 1.2 Hz, H-12). The latter showed a key HMBC correlation to C-10, indicating the formation of a new pyran ring based on the ether bond C-12–O–C-10. The relative configuration of **7** was assigned by the ROESY spectrum, in which the correlations of H-5/H-6a, H-6b/H-13, and H-13/H-11 suggested that H-5, OH-4, and OH-11 were on the same side. However, the stereoconfiguration of C-12 could not be determined by the ROESY spectrum. The small coupling constant ($^{3}J_{11,12}$ = 1.2 Hz) suggested that H-11 and H-12 should be axial and equatorial oriented, namely that OH-11 and OH-12 were in the opposite side. Accordingly, compound **7** was characterized and named as agrocybin G.

**Table 3.** $^1$**H NMR spectroscopic data (400 MHz) for compounds 4–7.**

| Position | **4**[a] | **5**[a] | **6**[b] | **7**[b] |
|---|---|---|---|---|
| 5 | 3.38, ddd (8.2, 8.0, 2.5) | 3.34, dd (8.1, 2.6) | 2.59, m | 3.55, dd (8.3, 2.7) |
| 6a | 1.97, dd (13.3, 8.0) | 1.97, dd (13.3, 5.2) | 1.55, m | 2.00, dd (13.4, 8.3) |
| 6b | 1.82, dd (13.3, 8.2) | 1.83, dd (13.3, 5.2) | 1.11, m | 1.86, dd (13.4, 8.3) |
| 8a | 6.52, d (2.5) | 6.52, d (2.6) | 2.22, dd (13.4, 2.3) | 6.53, d (2.7) |
| 8b | | | 1.52, m | |
| 9 | | | 2.93, m | |
| 10a | 1.84, s | 1.86, s | 5.15, s | 4.41, d (16.5) |
| 10b | | | | 4.20, d (16.5) |
| 11a | 2.93, ddd (13.3, 11.6, 5.1) | 2.77, m | 2.34, dd (19.1, 3.5) | 4.12, br. s |
| 11b | 2.55, ddd (13.3, 3.5, 3.0) | | 2.46, ddd (19.1, 11.4, 6.0) | |
| 12a | 4.00, ddd (9.5, 5.1, 3.0) | 4.48, dt (10.8, 7.4) | 3.81, dd (11.4, 6.0) | 5.14, d (1.2) |
| 12b | 3.80, ddd (11.6, 9.5, 3.5) | 4.21, dt (10.8, 6.9) | 3.91, ddd (11.4, 11.4, 3.5) | |
| 13 | 1.11, s | 1.13, s | 1.27, s | 1.15, s |
| 14 | 1.11, s | 1.12, s | 0.95, s | 1.14, s |
| 15 | 1.20, s | 1.20, s | 1.04, s | 1.22, s |
| $OCH_3$ | | | 3.40, s | |
| $CH_3CO$ | | 2.04, s | | |

[a]spectra were measured in $CDCl_3$; [b]spectra were measured in methanol-$d_4$.

All the isolated compounds were tested for their cytotoxicity against five human cancer cell lines (HL-60, SMMC-7712, A-549, MCF-7, and SW480) by the MTT method, with DDP and taxol as positive controls. Unfortunately, the result showed that none of the compounds exhibited obvious activity at the concentration of 40 $\mu$M. Surveys on the literature revealed that the coexistence of a cyclopropane-ring and a leaving group (such as hydroxy) at C-2 or C-4 are necessary for illudins to possess cytotoxicities.[18–20] However, all the tested compounds do not meet the above structural features.

## Experimental Section

**General Experimental Procedures**. Melting points were surveyed with an X-4 microscopic melting point meter. Optical rotations were measured on a Horiba SEPA-300 spectropolarimeter. UV spectra were recorded on a Shimadzu double-beam 210A spectrometer. IR spectra were obtained on a Bruker Tensor 27 FT-IR spectrometer using KBr pellets. NMR spectra were acquired on Bruker DRX-500 and AM-400 instruments at room temperature with TMS as an internal standard. Chemical shifts ($\delta$) were expressed in ppm with reference to the solvent signals. Mass spectra (MS) were recorded on an API QSTAR time-of-flight spectrometer or a VG Autospec-3000 spectrometer. X-ray crystallographic data were collected on a Bruker APEX DUO diffractometer with graphite-monochromated Mo Kα radiation. Silica gel (200–300 mesh, Qingdao Marine Chemical Inc., China), Sephadex LH-20 (Amersham Biosciences, Sweden), and RP-18 gel (40–75 $\mu$m, Fuji Silysia Chemical Ltd. Japan) were used for column chromatography (CC). Preparative HPLC (Prep-HPLC) was performed on an Agilent 1100 liquid chromatography system equipped with a Zorbax SB-$C_{18}$ column (9.4 mm × 150 mm). Pre-coated silica gel GF254 plates (Qingdao Marine Chemical Inc., China) were used for TLC. Fractions were monitored by TLC, and spots were visualized by heating silica gel plates sprayed with 10% $H_2SO_4$ in ethanol.

**Fungal Material and Cultivation Conditions.** The fungus *A. salicacola* was collected at the Botanic Garden of Kunming Institute of Botany, Chinese Academy of Sciences, China, in spring 2008, and identified by Prof. Mu Zang, Kunming Institute of Botany. The voucher specimen has been deposited in the Herbarium of Kunming Institute of Botany, Chinese Academy of sciences. The liquid culture medium contained saccharine 5%, yeast powder 0.5%, peptone 0.15%, $KH_2PO_4$ 0.05%, and $MgSO_4$ 0.05%. Inoculums of *A. salicacola* were prepared in a 15 L-fermentor (Biostar, Shanghai GuoQiang, China) for 6 days under the following conditions: culture temperature 24 °C, initial pH 6.0, agitation speed 250 r/min, inoculation volume 10% (by volume), and aeration rate 1.0 vvm. Then, the liquid seed was transferred into a 100 L-fermentation tank to be cultivated under the same conditions for 20 days to afford 80 L culture broth.

**Extraction and Isolation.** The entire culture broth of *A. salicacola* (80 L) was initially filtered, and the filtrate was extracted three times with EtOAc. The organic layer was concentrated under reduced pressure to give a crude extract (280 g), and this residue was subjected to CC over silica gel using a petroleum ether-$Me_2CO$ gradient (1:0 → 0:1) to afford fractions A-L. Fraction B was subjected to CC over silica gel eluted with a petroleum ether-$Me_2CO$ system (20:1) to get **1** (4 mg). Fraction D was first isolated by CC over silica gel (petroleum ether-$Me_2CO$) and Sephadex LH-20 (MeOH), and then purified by Prep-HPLC (MeCN/$H_2O$, 3:7 → 5:5) to give **5** (5.0 mg). Fraction F (65 g) was subjected to CC over silica gel to provide four subfractions ($F_1$–$F_4$). Fraction $F_2$ was first separated by silica gel CC (petroleum ether-$Me_2CO$, 5:1), then purified by Prep-HPLC (MeCN/$H_2O$, 2:8 → 5:5) to afford **2** (4.0 mg), **3** (1.4 mg), **9** (8.0 mg), and **10** (9.0 mg). Compounds **6** (4.0 mg) and **7** (7.0 mg) were obtained from fraction $F_3$ by Prep-HPLC (MeCN/$H_2O$, 2:8 → 4:6). Fraction I (36 g) was subjected to Sephadex LH-20 ($CHCl_3$-MeOH, 1:1), and then purified by the RP-18 CC (MeOH/$H_2O$, 4:6) to yield **4** (5.0 mg). Fraction J (10 g) was subjected to silica gel CC eluted using a $CH_3Cl$-MeOH gradient (15:1 → 5:1), and then purified by Prep-HPLC (MeCN/$H_2O$, 3:7) to afford **8** (12.5 mg).

**Agrocybin A (1):** colorless needles (acetone); mp 139–140 °C; $[\alpha]_D^{24} = -69.0$ (*c* 0.24, $CHCl_3$); IR (KBr) $\nu_{max}$ 3425, 2945, 2837, 1112, 1059, 911 $cm^{-1}$; $^{13}C$ NMR data, see Table 1; $^1H$ NMR data, see Table 2; ESIMS (positive) *m/z* 287 [M + Na]$^+$; HRESIMS (positive) *m/z* 287.1251 (calcd. for $C_{15}H_{20}O_4Na$, 287.1259).

**Agrocybin B (2):** colorless oil; $[\alpha]_D^{16} - 14.7$ (*c* 0.38, $CHCl_3$); IR (KBr) $\nu_{max}$ 3439, 1631 $cm^{-1}$; $^{13}C$ NMR data, see Table 1; $^1H$ NMR data, see Table 2; ESIMS (positive) *m/z* 237 [M + H]$^+$; HRESIMS (positive) *m/z* 259.1676 [M + Na]$^+$ (calcd. for $C_{15}H_{24}O_2Na$, 259.1673).

**Agrocybin C (3):** colorless oil; $[\alpha]_D^{12} - 11.0$ (*c* 0.10, MeOH); IR (KBr) $\nu_{max}$ 3431, 2955, 1721, 1632, 1172 $cm^{-1}$; $^{13}C$ NMR data, see Table 1; $^1H$ NMR data, see Table 2; ESIMS (positive) *m/z* 287 [M + Na]$^+$; HRESIMS (positive) *m/z* 287.1626 [M + Na]$^+$ (calcd. for $C_{16}H_{24}O_3Na$, 287.1623).

**Agrocybin D (4):** colorless oil; $[\alpha]_D^{13} - 75.2$ (*c* 0.26, MeOH); UV (MeOH) $\lambda_{max}$ (log $\varepsilon$) 286 (3.09), 261 (3.11), 204 (2.98), 194 (2.79) nm; IR (KBr) $\nu_{max}$ 3423, 2957, 1641, 1625 $cm^{-1}$; $^{13}C$ NMR data, see Table 1; $^1H$ NMR data, see Table 3; ESIMS (positive) *m/z* 251 [M + H]$^+$; HRESIMS (positive) *m/z* 251.1649 [M + H]$^+$ (calcd. for $C_{15}H_{23}O_3$, 251.1647).

**Agrocybin E (5):** colorless oil; $[\alpha]_D^{21} - 93.4$ (*c* 0.32, $CHCl_3$); IR (KBr) $\nu_{max}$ 3449, 2957, 1742, 1642, 1626, 1238 $cm^{-1}$; $^{13}C$ NMR data, see Table 1; $^1H$ NMR data, see Table 3; ESIMS (positive) *m/z* 315 [M + Na]$^+$; HRESIMS (positive) *m/z* 315.1566 [M + Na]$^+$ (calcd. for $C_{17}H_{24}O_4Na$, 315.1572).

**Agrocybin F (6):** colorless needles (acetone-methanol); mp 168–169 °C; $[\alpha]_D^{13} + 65.4$ (*c* 0.18, MeOH); UV (MeOH) $\lambda_{max}$ (log $\varepsilon$) 233 (3.06) nm; IR (KBr) $\nu_{max}$ 3465, 2955, 2924, 1656, 1062 $cm^{-1}$; $^{13}C$ NMR data, see Table 1; $^1H$ NMR data, see Table 3; ESIMS (positive) *m/z* 303 [M + Na]$^+$; HRESIMS (positive) *m/z* 303.1580 [M + Na]$^+$ (calcd. for $C_{16}H_{24}O_4Na$, 303.1572).

**Agrocybin G (7):** colorless oil; $[\alpha]_D^{13} - 94.8$ (*c* 0.15, MeOH); UV (MeOH) $\lambda_{max}$ (log $\varepsilon$) 207 (3.14), 249 (2.91) nm; IR (KBr) $\nu_{max}$ 3440, 2957, 2933, 1630 $cm^{-1}$; $^{13}C$ NMR data, see Table 1; $^1H$ NMR data, see Table 3; ESIMS (positive) *m/z* 303 [M + Na]$^+$; HRESIMS (positive) *m/z* 303.1204 [M + Na]$^+$ (calcd. for $C_{15}H_{20}O_5Na$, 303.1208).

**Crystallographic Data of Agrocybin A (1):** $C_{15}H_{20}O_4$; $M$ = 264.31; trigonal; space group P3$_1$; $a = b = 12.686$ (3) Å, $c$ = 7.2090 (16)Å; $\alpha = \beta = 90°$, $\gamma = 120°$; $V$ = 1004.8 (4) Å$^3$; $Z$ = 3; $\rho$ = 1.310 g·cm$^{-3}$; crystal dimensions 0.05×0.07×0.64 mm$^3$; Shelxs97 with a graphite monochromator; Mo K$\alpha$ radiation. The total number of reflections measured was 14186, of which 3002 were observed, $I > 2\sigma(I)$; $R_1$ = 0.0408, $wR_2$ = 0.0914. Crystallographic data for agrocybin A (**1**) has been deposited at the Cambridge Crystallographic Data Centre as deposition number CCDC 842502. Copies of the data can be obtained free of charge on application to CCDC, 12 Union Road, Cambridge CB2 1EZ, UK (Tel: +44 (0)1223 762911, e-mail: deposit@ccdc.cam.ac.uk).

**Crystallographic Data of Agrocybin F (6):** $C_{16}H_{24}O_4$; $M$ = 280.35; orthorhombic; space group P2$_1$2$_1$2$_1$; $a$ = 6.1050(6), $b$ = 13.9122(13), $c$ = 17.2488(16)Å; $\alpha = \gamma = \beta = 90°$; $V$ = 1465.0(2)Å$^3$; $Z$ = 4; $\rho$ = 1.271 g·cm$^{-3}$; crystal dimensions 0.11×0.43×0.87 mm$^3$; Shelxs97 with a graphite monochromator; Mo K$\alpha$ radiation. The total number of reflections measured was 14233, of which 3436 were observed, $I > 2\sigma(I)$; $R_1$ = 0.0279, $wR_2$ = 0.0754. Crystallographic data for agrocybin F (**6**) has been deposited at the Cambridge Crystallographic Data Centre as deposition number CCDC 842503. Copies of the data can be obtained free of charge on application to CCDC, 12 Union Road, Cambridge CB2 1EZ, UK (Tel: +44 (0)1223 762911, e-mail: deposit@ccdc.cam.ac.uk).

**Cytotoxicity Assay.** The following human tumor cell lines were used: HL-60, SMMC-7712, A-549, MCF-7, and SW480. All the cells were cultured in RMPI-1640 or DMEM medium (Hyclone, Logan, UT), supplemented with 10% fetal bovine serum (Hyclone) at 37 °C in a humidified atmosphere with 5% $CO_2$. Cell viability was assessed by conducting colorimetric measurements of the amount of insoluble formazan formed in living cells based on the reduction of 3-(4,5-dimethylthiazol-2-yl)-2,5-diphenyltetrazolium bromide (MTT) (Sigma, St. Louis, MO). Briefly, 100 $\mu$L of adherent cells were seeded into each well of a 96-well cell culture plate and allowed to adhere for 12 h before drug addition, while suspended cells were seeded just before drug addition, both with an initial density of 1 × 10$^5$ cells/mL in 100 $\mu$L of medium. Each tumor cell line was exposed to the test compounds at various concentrations in triplicate for 48 h, with DDP and toxal as positive controls. After the incubation, MTT (100 $\mu$g) was added to each well, and the incubation continued for 4 h at 37 °C. The cells lysed with 200 $\mu$L SDS after removal of 100 $\mu$L of medium. The optical density of lysate was measured at 595 nm in a 96-well microtiter plate reader (Bio-Rad 680). The $IC_{50}$ value of each compound was calculated by Reed and Muench's method.[21]

## Electronic Supplementary Material

Supplementary material is available in the online version of this article at http://dx.doi.org/10.1007/s13659-011-0018-4 and is accessible for authorized users.

## Acknowledgments

This project was supported by the National Basic Research Program of China (973 Program, 2009CB522300), the National Natural Sciences Foundation of China (30830113), and MOST (2009ZX09501-029).

**Open Access** This article is distributed under the terms of the Creative Commons Attribution License which permits any use, distribution, and reproduction in any medium, provided the original author(s)

and source are credited.

## Electronic supplementary material

Supplementary material, approximately 11.2 MB.
