# Supplementary material for: Unusual illudin-type sesquiterpenoids from cultures of Agrocybe salicacola
Source: Nat Prod Bioprospect. 2011 Oct 30;1(2):87–92. doi: 10.1007/s13659-011-0018-4 (PMC4131650; doi:10.1007/s13659-011-0018-4)

**Electronic Supplementary Material**

# Unusual illudin-type sesquiterpenoids from cultures of *Agrocybe salicacola*

Liang-Yan LIU,[a,b] Ling ZHANG,[a] Tao FENG,[a] Zheng-Hui LI,[a] Ze-Jun DONG,[a] Xing-Yao LI,[a,b] Jia SU,[a,b] Yan LI,[a] and Ji-Kai LIU[a,]*

[a]State Key Laboratory of Phytochemistry and Plant Resources in West China, Kunming Institute of Botany, Chinese Academy of Sciences, Kunming 650201, China

[b]Graduate University of Chinese Academy of Sciences, Beijing 100039, China

Received 19 September 2011; Accepted 26 ctober 2011

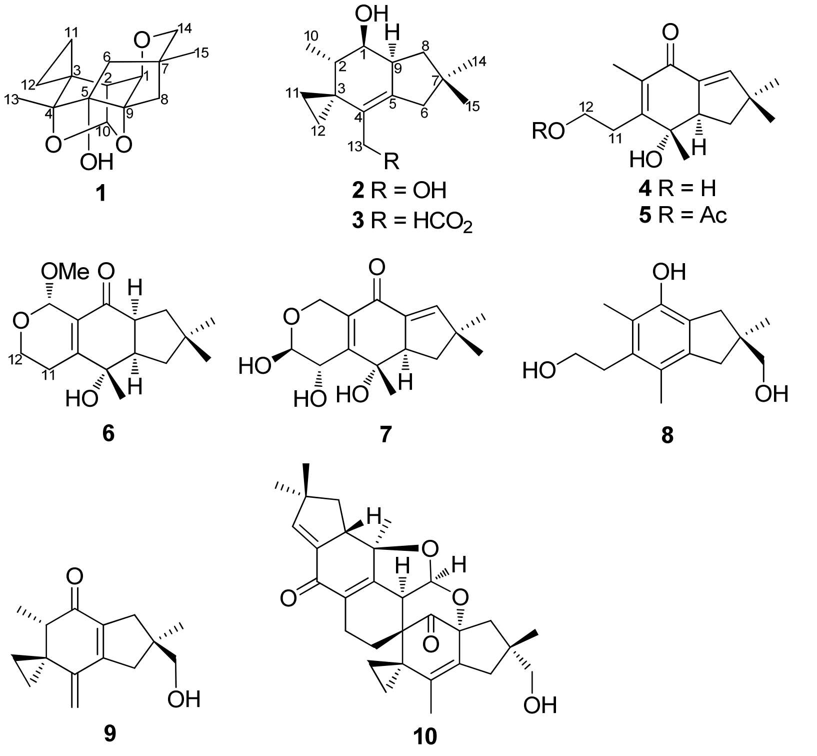


Structures of compounds **1**–**10**.

*To whom correspondence should be addressed. E-mail: jkliu@mail.kib.ac.cn.

## Table of Contents

1D and 2D NMR spectra of agrocybin A **(1)** S3

1D and 2D NMR spectra of agrocybin B **(2)** S6

1D and 2D NMR spectra of agrocybin C **(3)** S8

1D and 2D NMR spectra of agrocybin D **(4)** S11

$^{1}$H and $^{13}$C NMR spectra of agrocybin E **(5)** S14

1D and 2D NMR spectra of agrocybin F **(6)** S16

1D and 2D NMR spectra of agrocybin G **(7)** S18

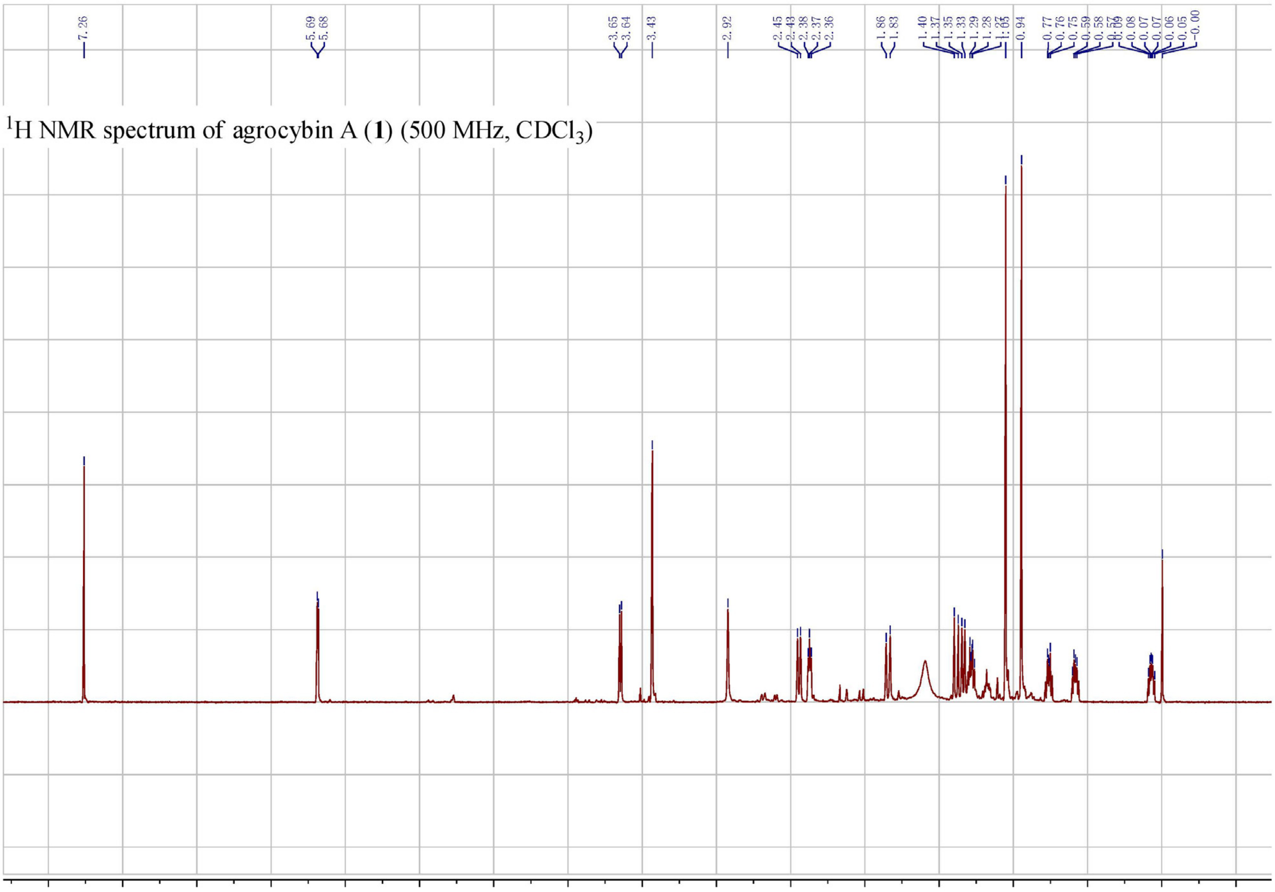


$^1$H NMR spectrum of agrocybin A (**1**) (500 MHz, $CDCl_3$)

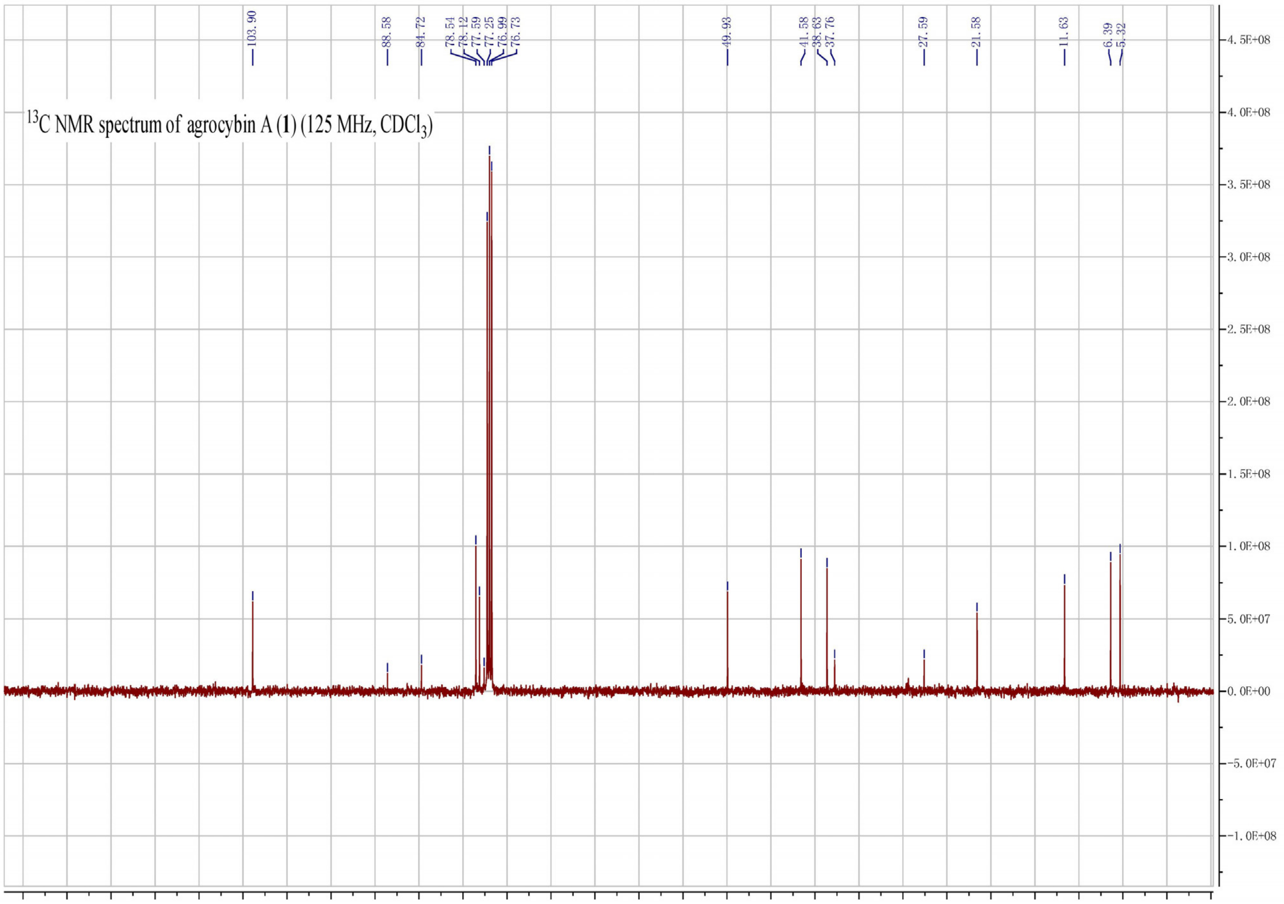


$^{13}$C NMR spectrum of agrocybin A (**1**) (125 MHz, $CDCl_3$)

HMBC spectrum of agrocybin A (**1**)

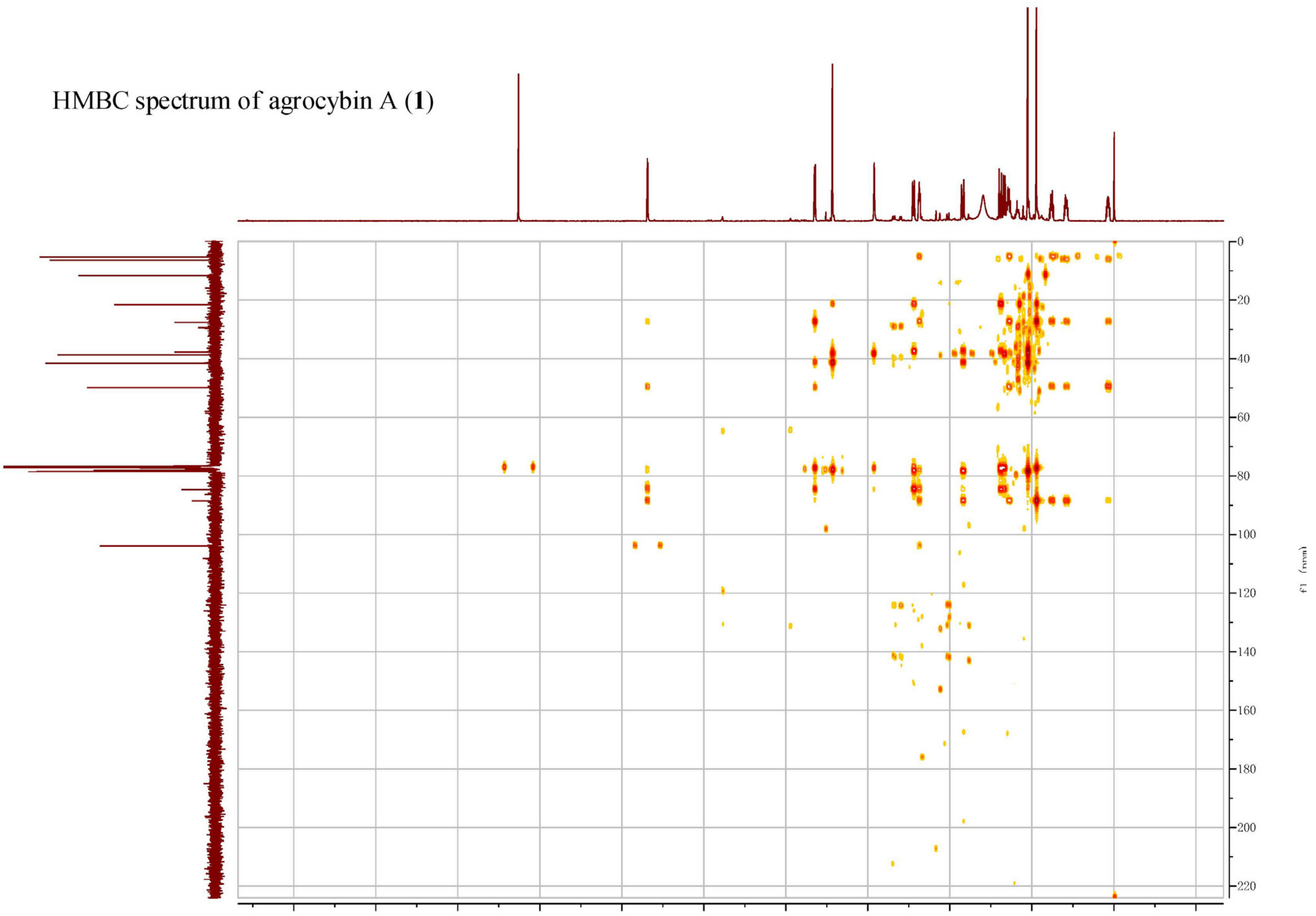


HSQC spectrum of agrocybin A (**1**)

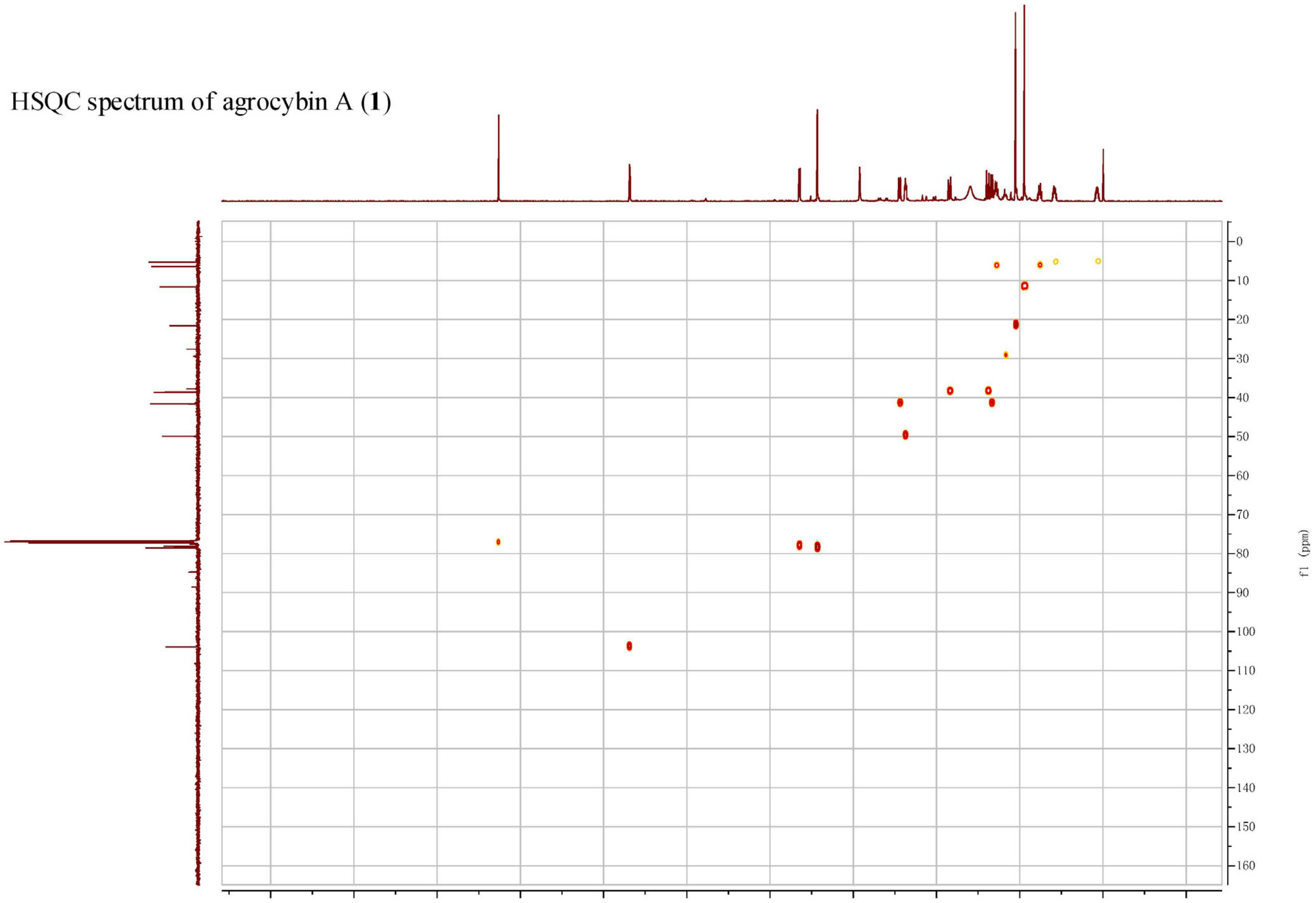

COSY spectrum of agrocybin A (**1**)

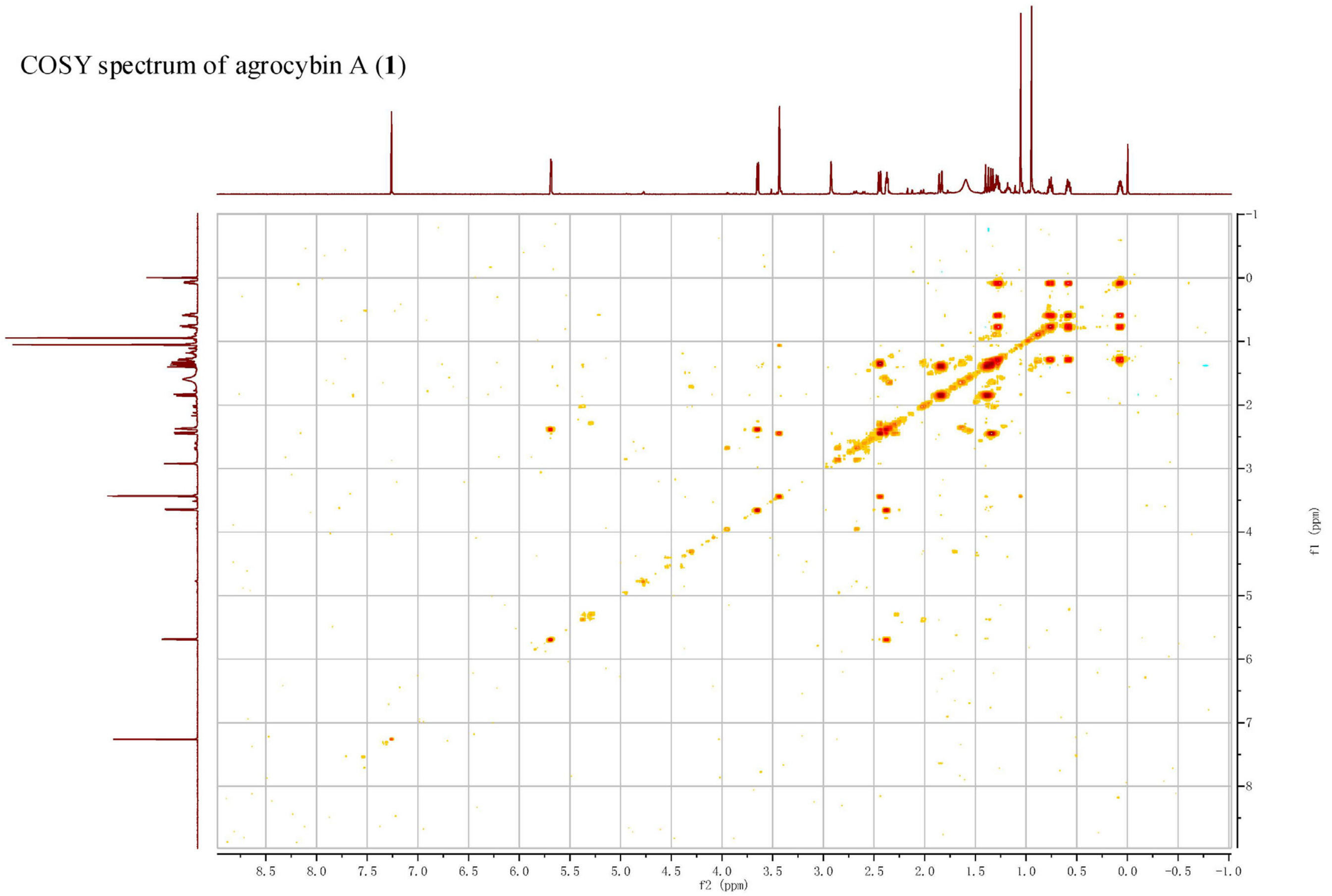


ROESY spectrum of agrocybin A (**1**)

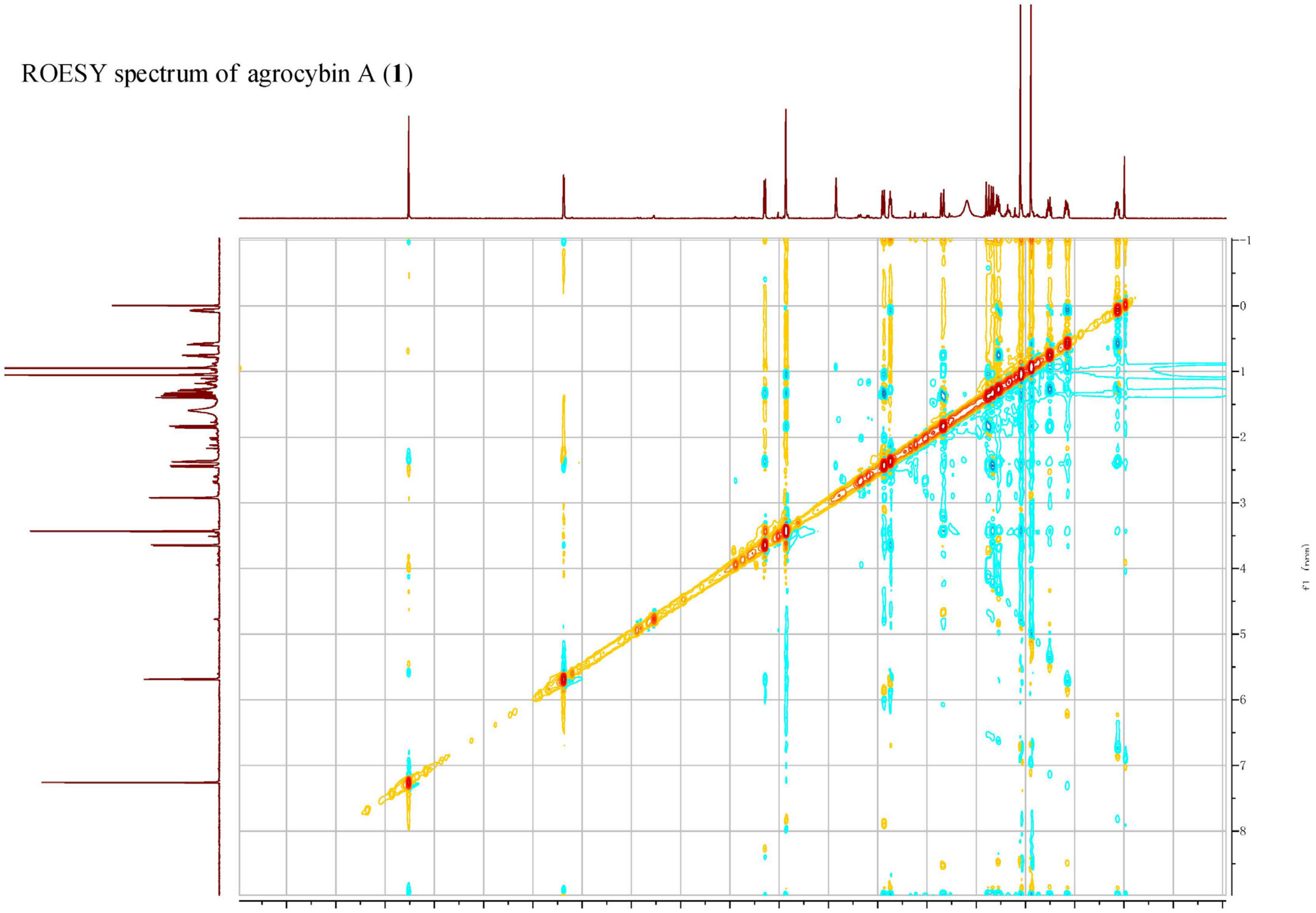

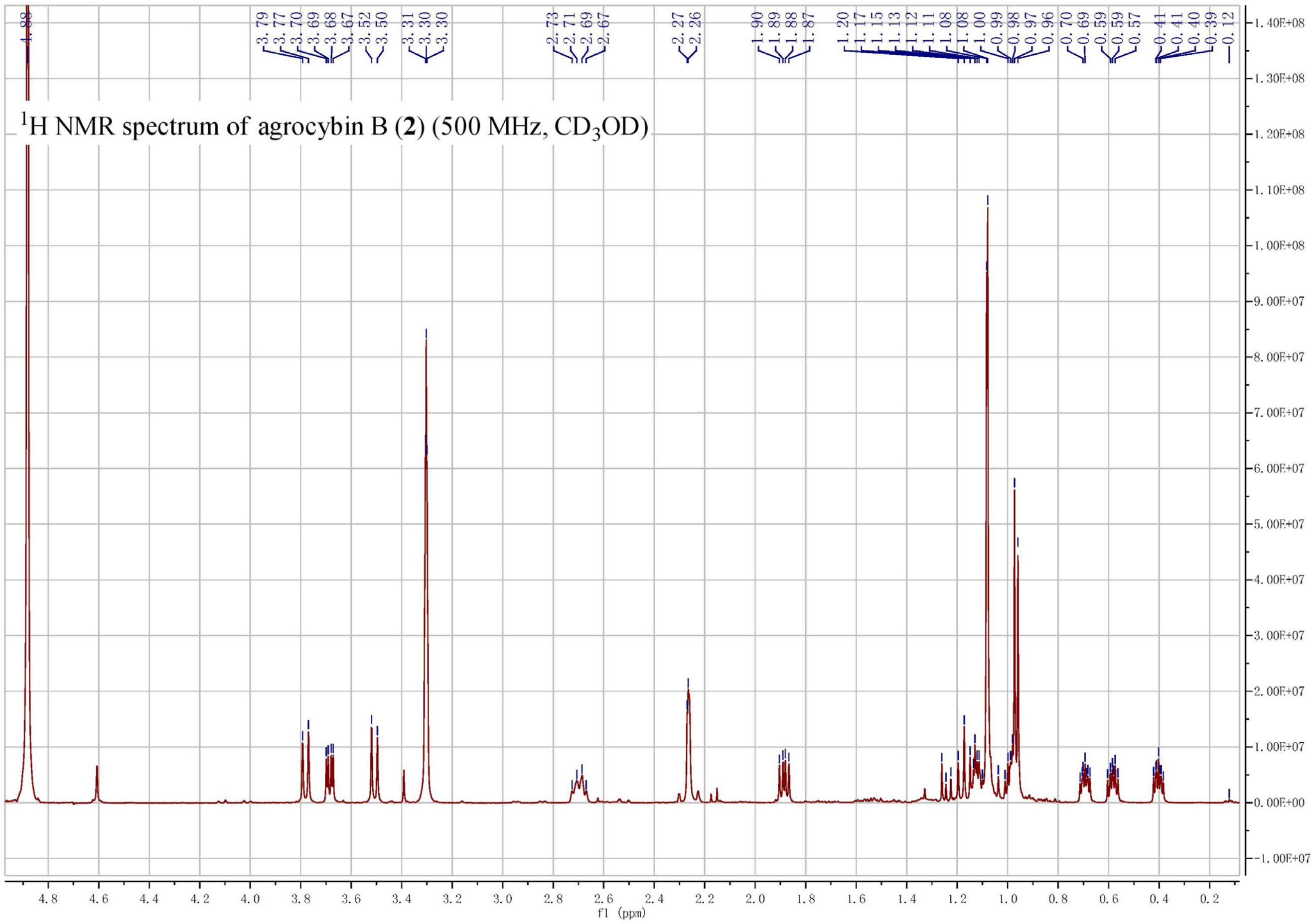

$^{1}$H NMR spectrum of agrocybin B (2) (500 MHz, CD3OD)
f1 (ppm)


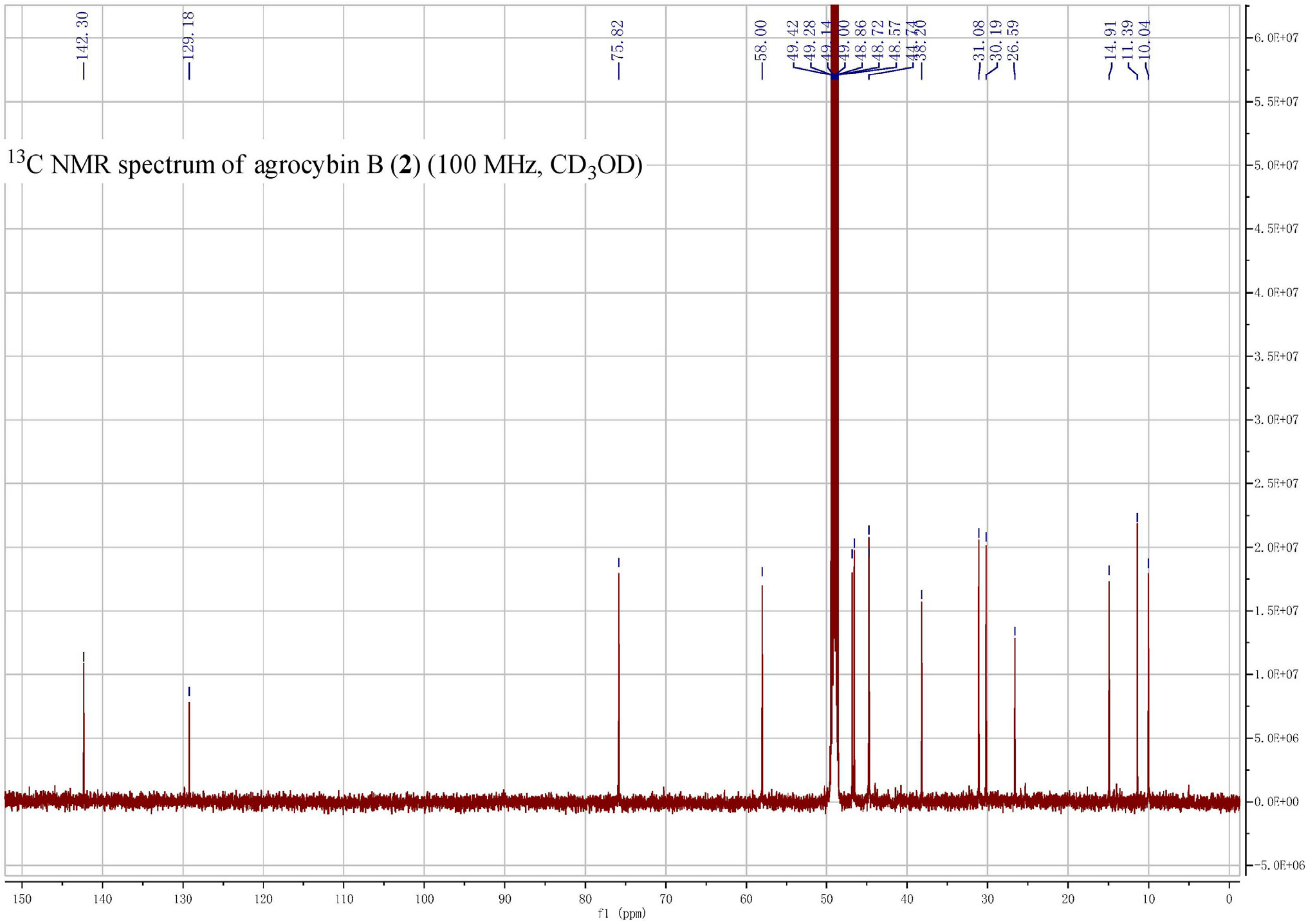

$^{13}$C NMR spectrum of agrocybin B (2) (100 MHz, CD3OD)
f1 (ppm)

HMBC spectrum of agrocybin B (**2**)

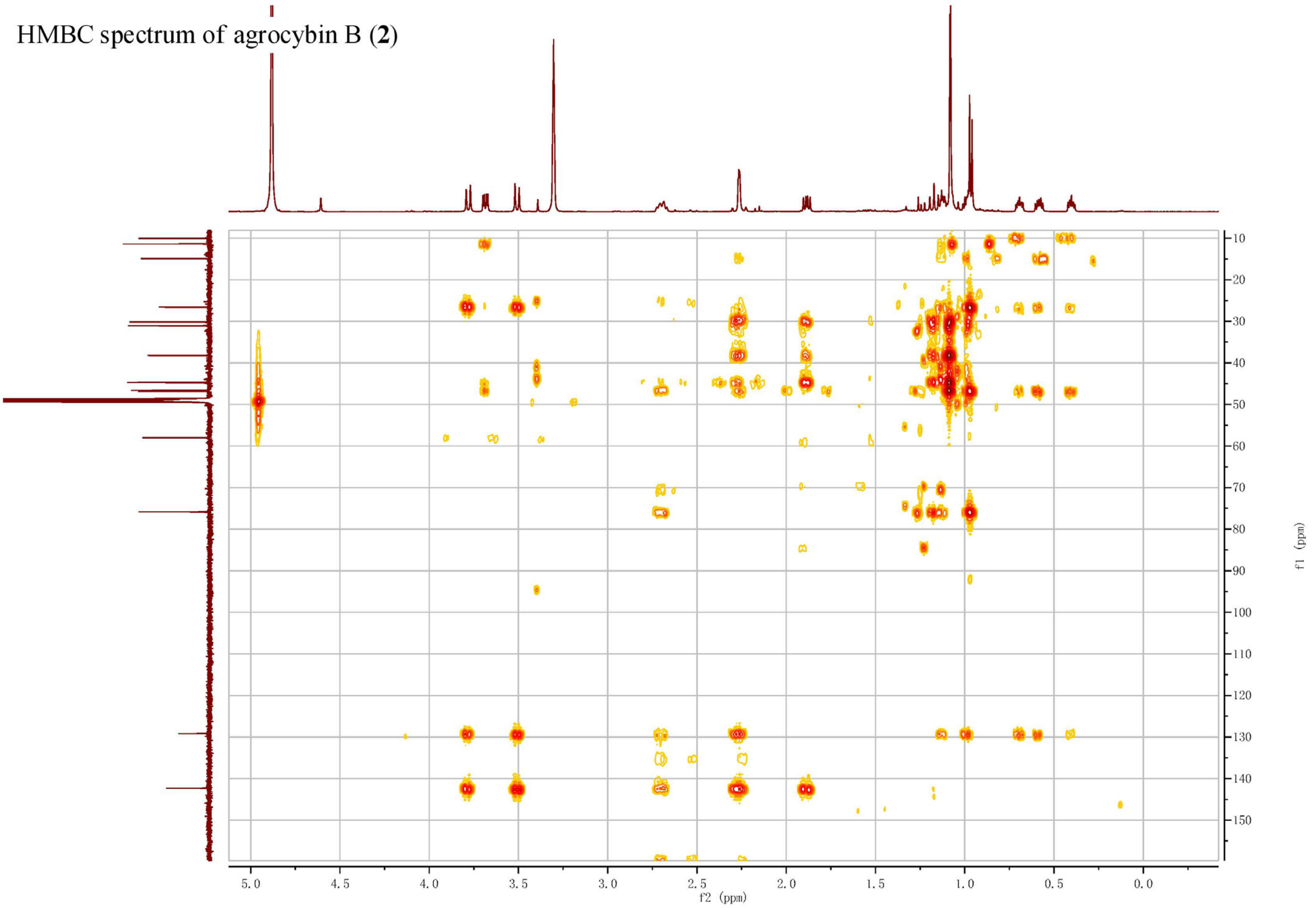


HSQC spectrum of agrocybin B (**2**)

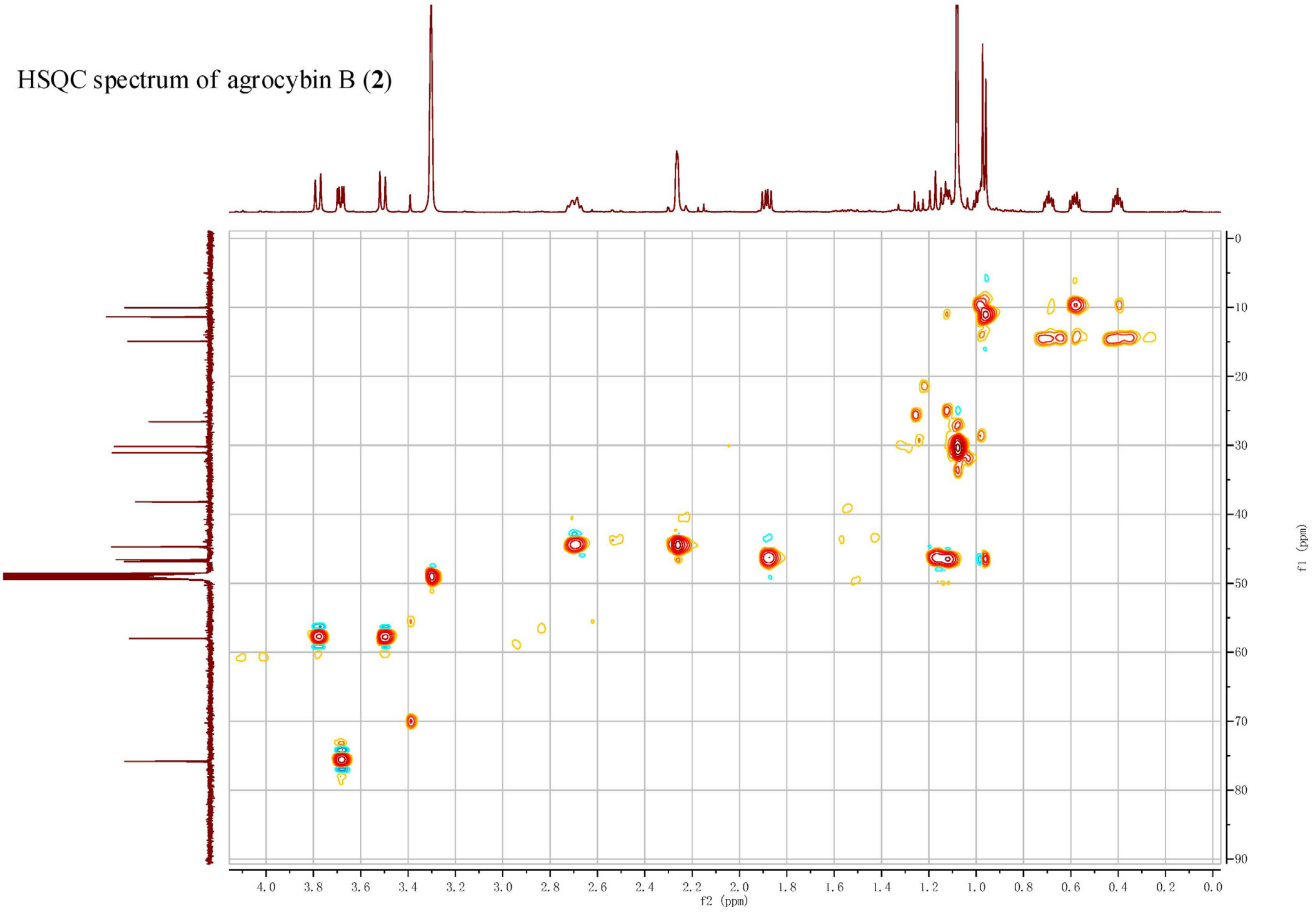

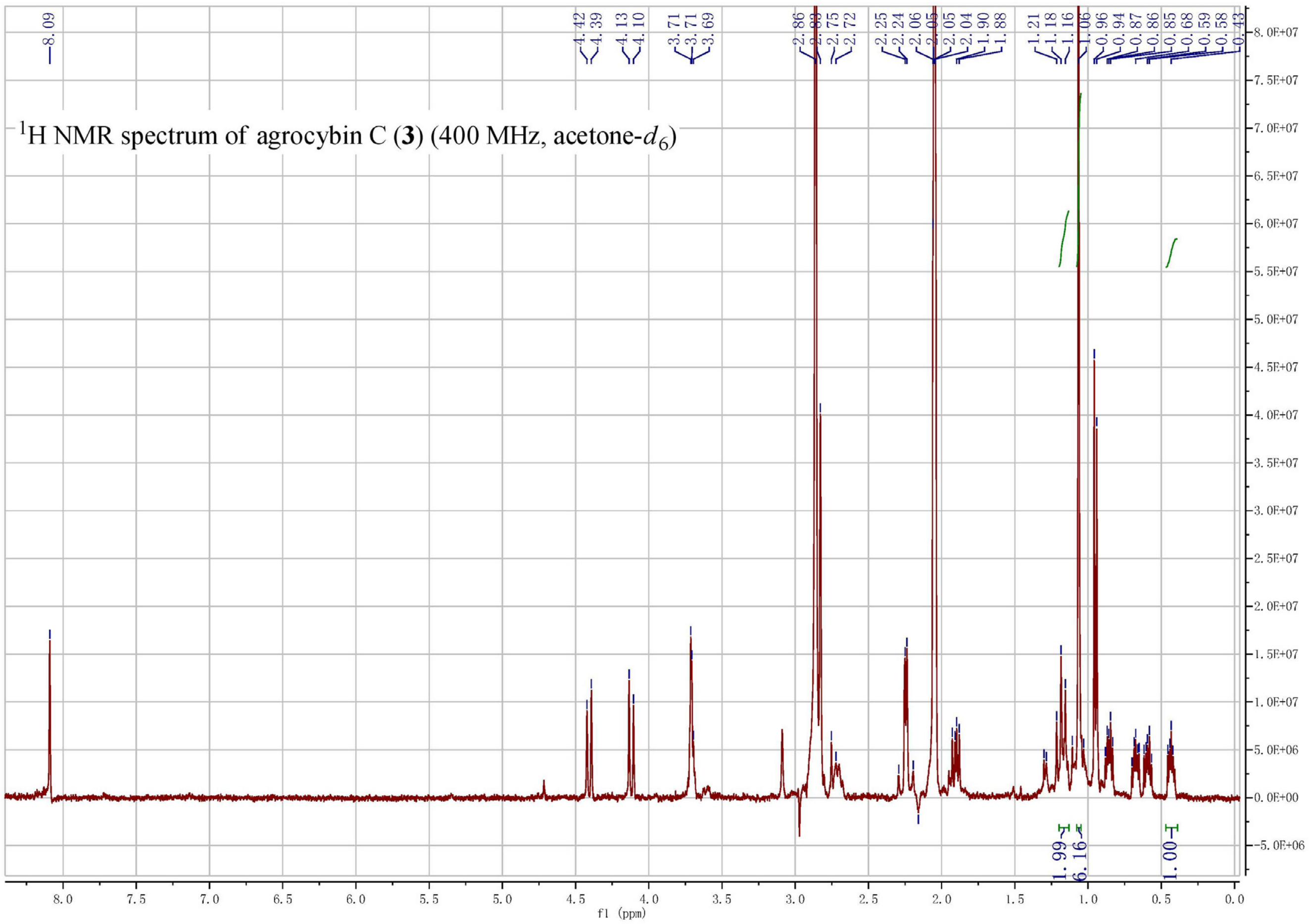
$^{1}$H NMR spectrum of agrocybin C (**3**) (400 MHz, acetone-$d_6$)

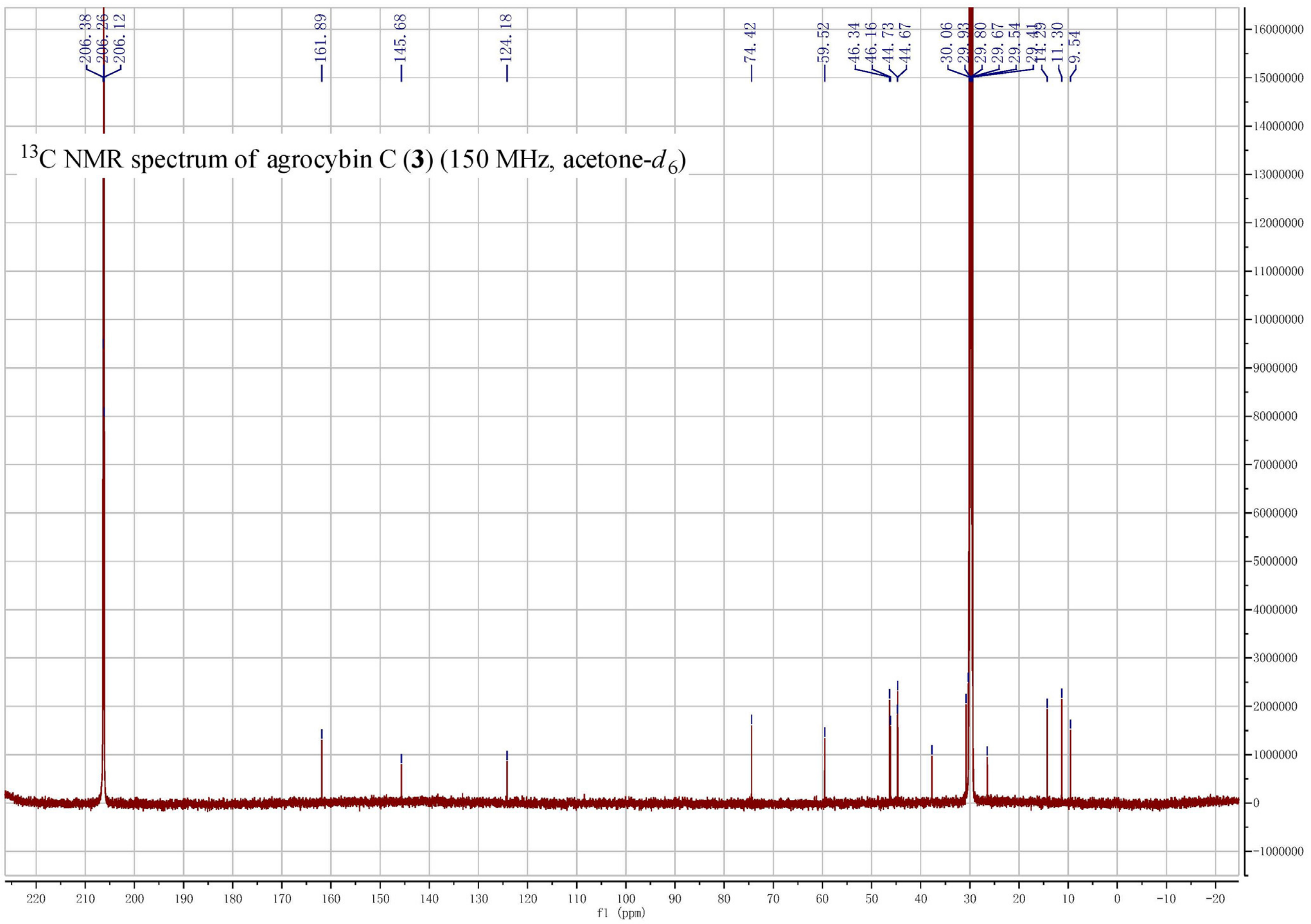
$^{13}$C NMR spectrum of agrocybin C (**3**) (150 MHz, acetone-$d_6$)

HSQC spectrum of agrocybin C (**3**)

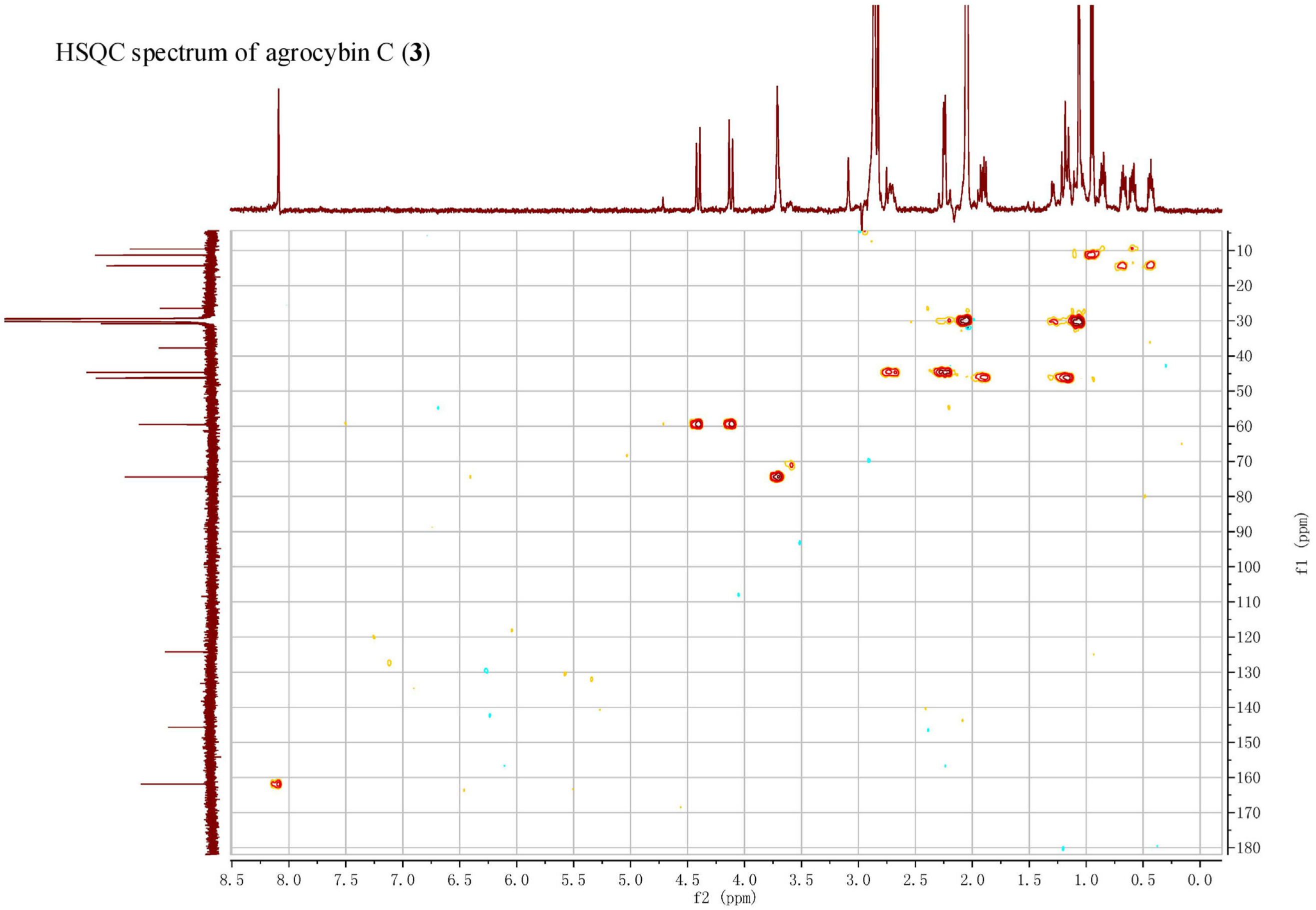


HMBC spectrum of agrocybin C (**3**)

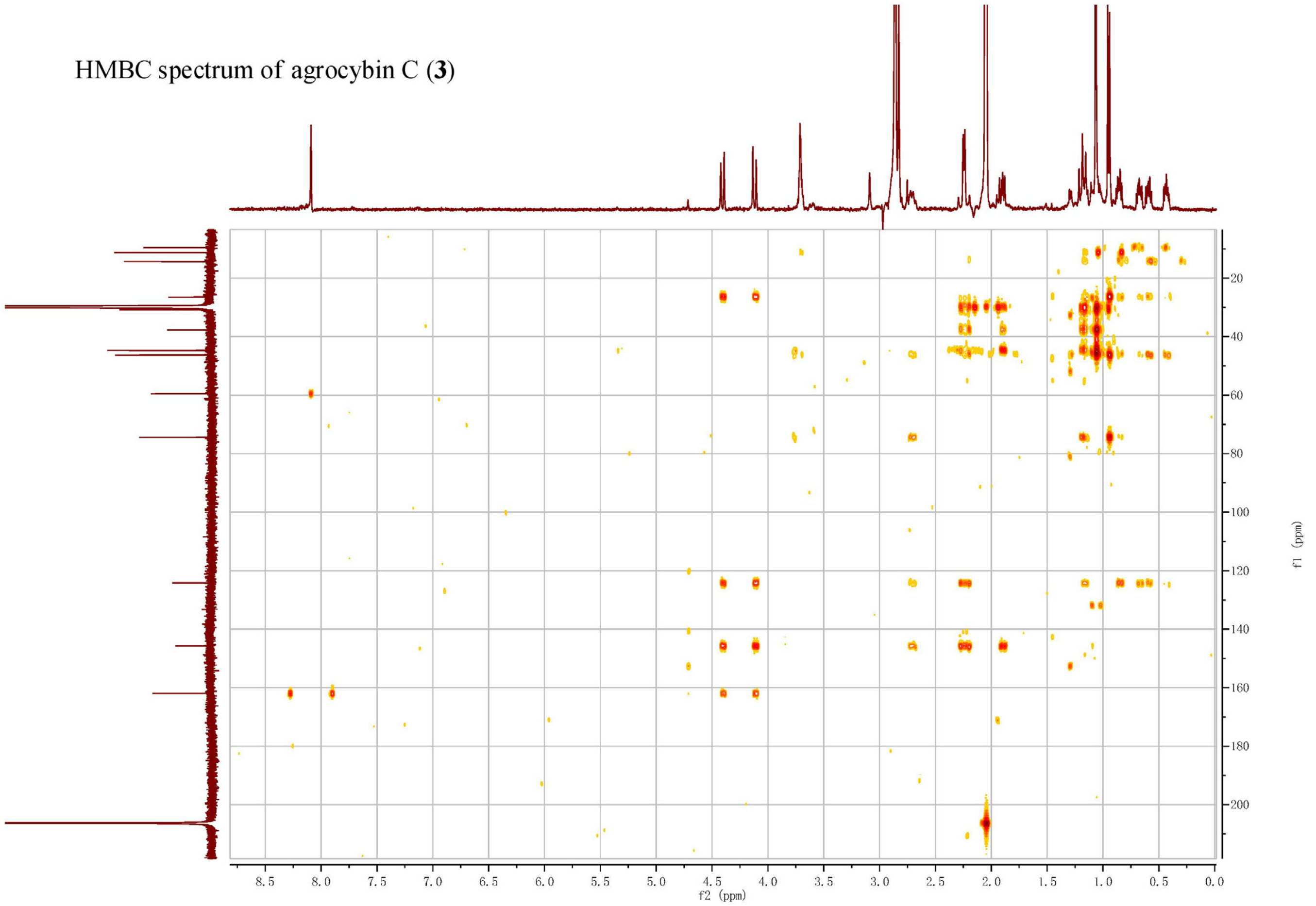

ROESY spectrum of agrocybin C (**3**)

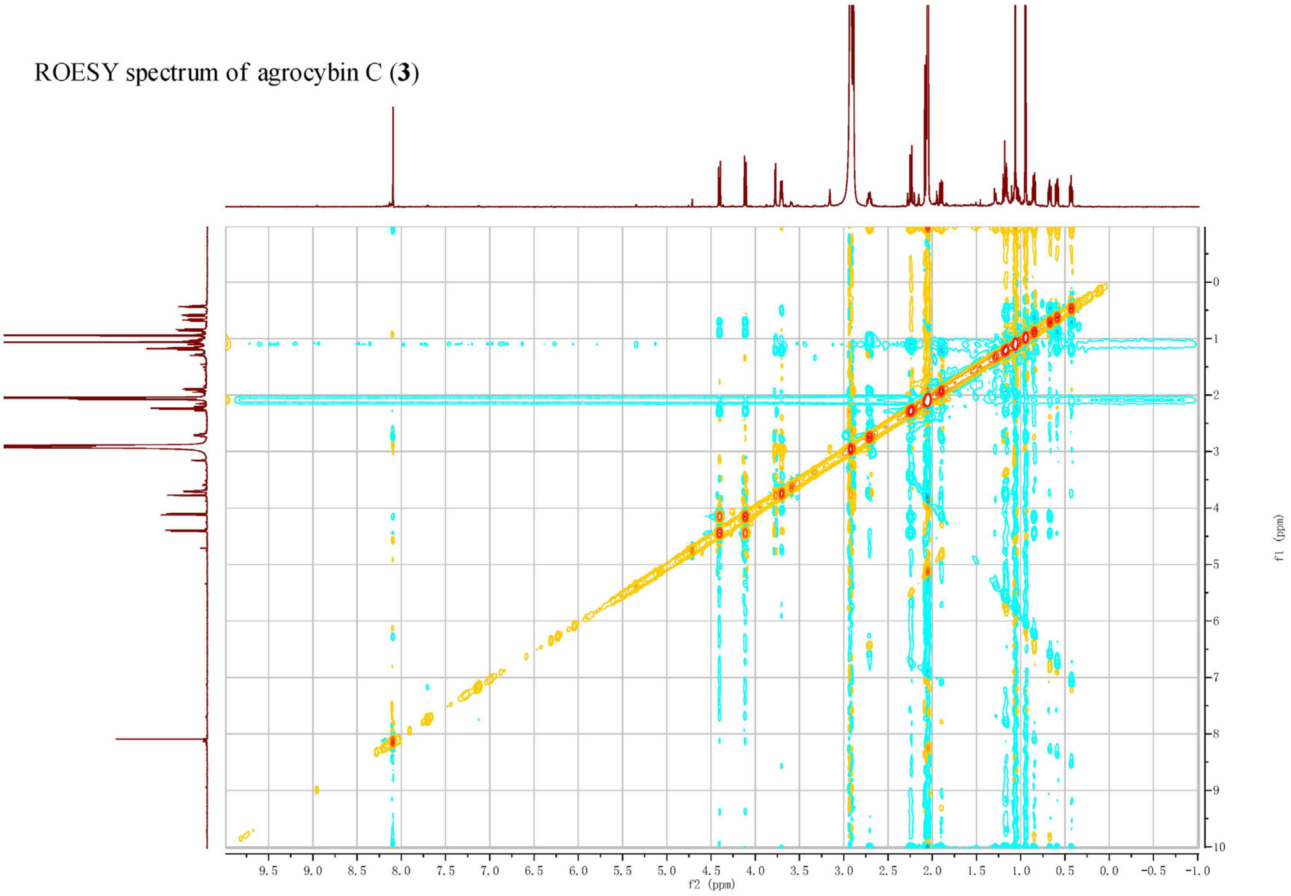

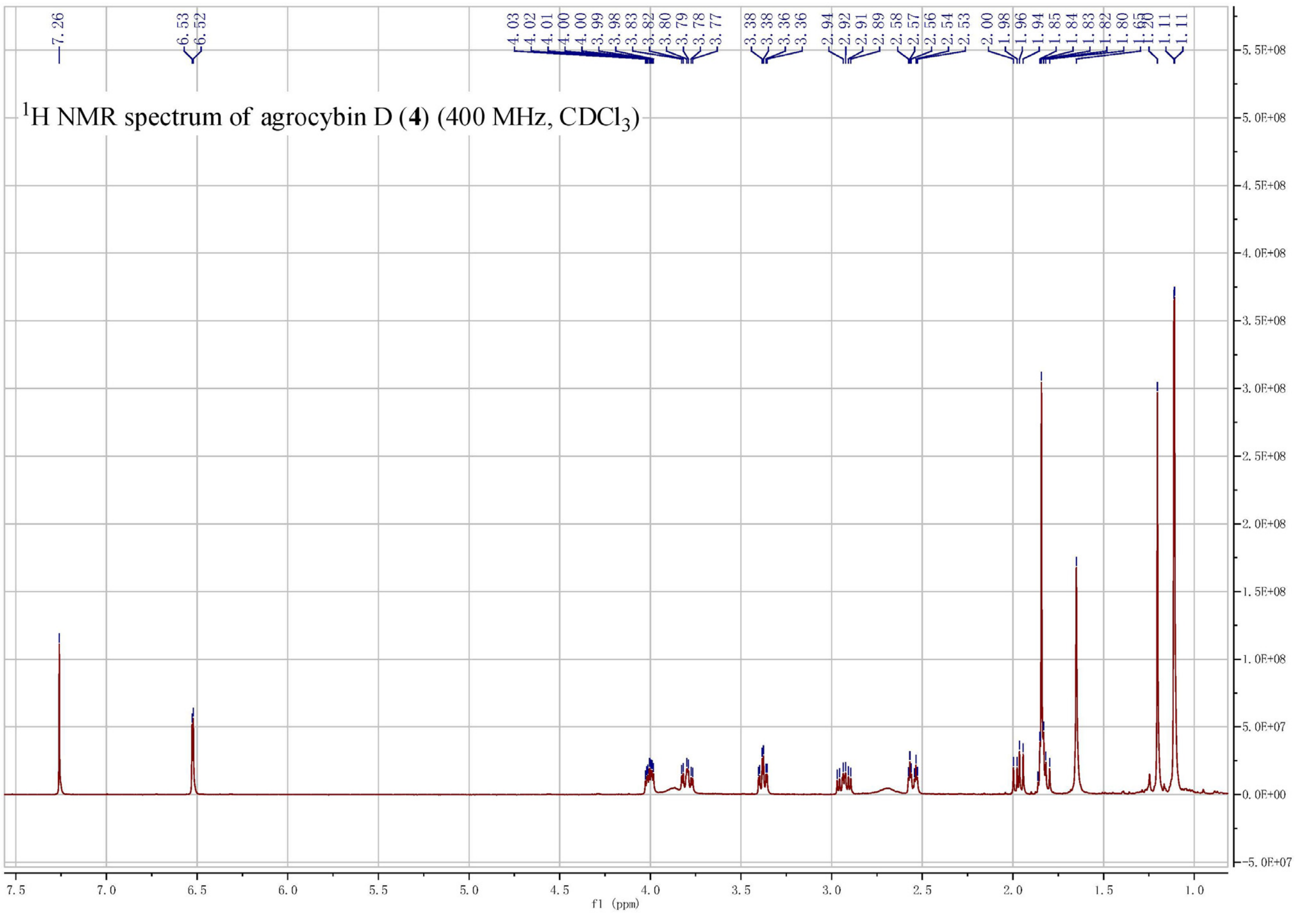
$^{1}$H NMR spectrum of agrocybin D (**4**) (400 MHz, $CDCl_3$)

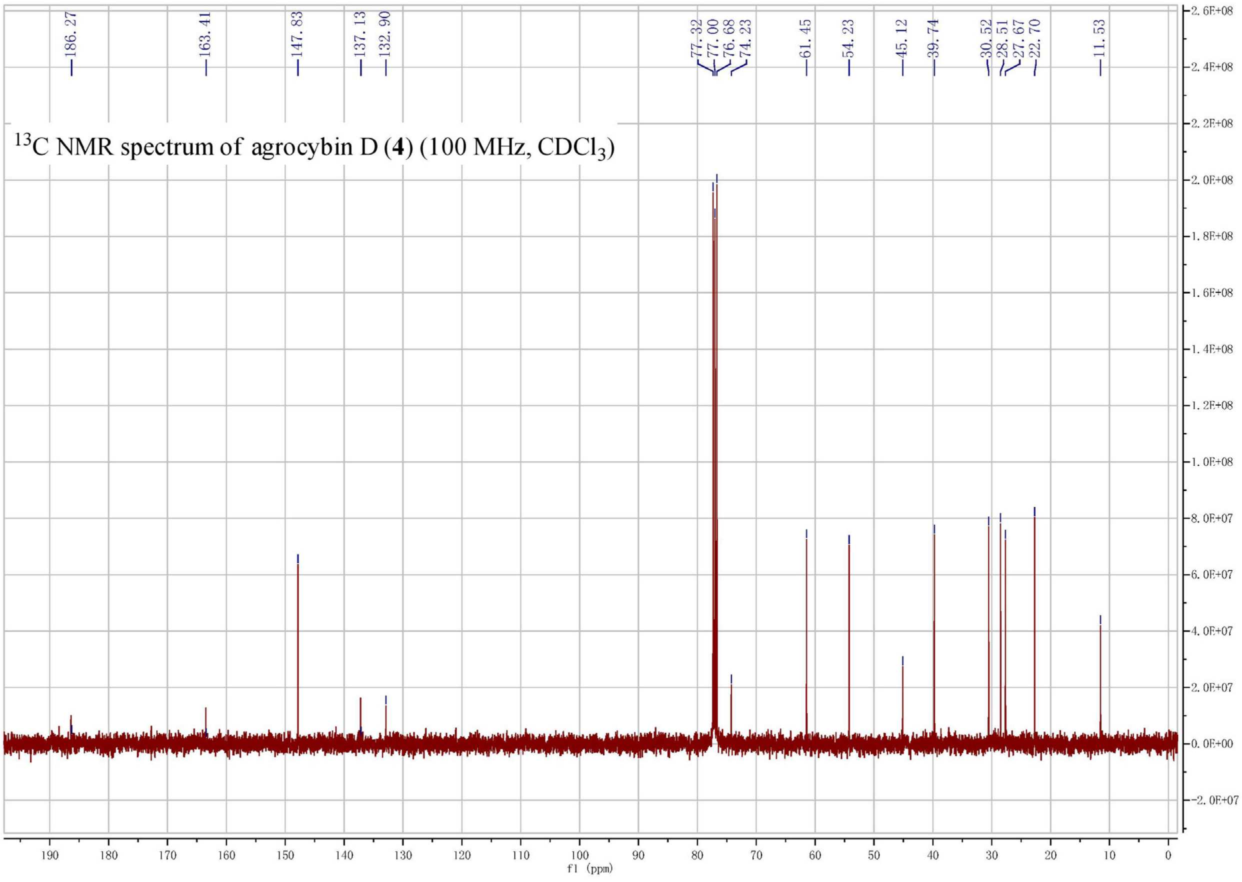
$^{13}$C NMR spectrum of agrocybin D (**4**) (100 MHz, $CDCl_3$)

HSQC spectrum of agrocybin D (**4**)

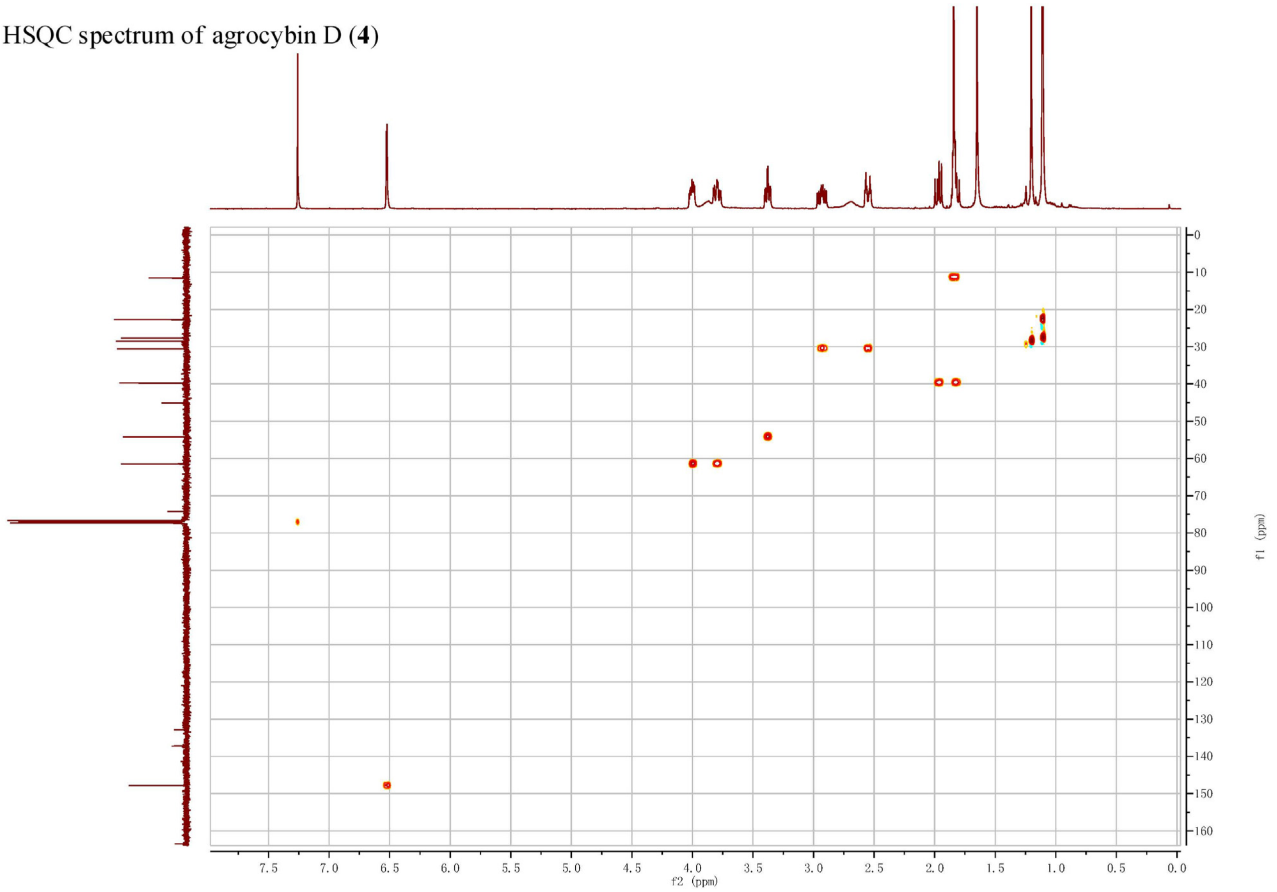


HMBC spectrum of aAgrocybin D (**4**)

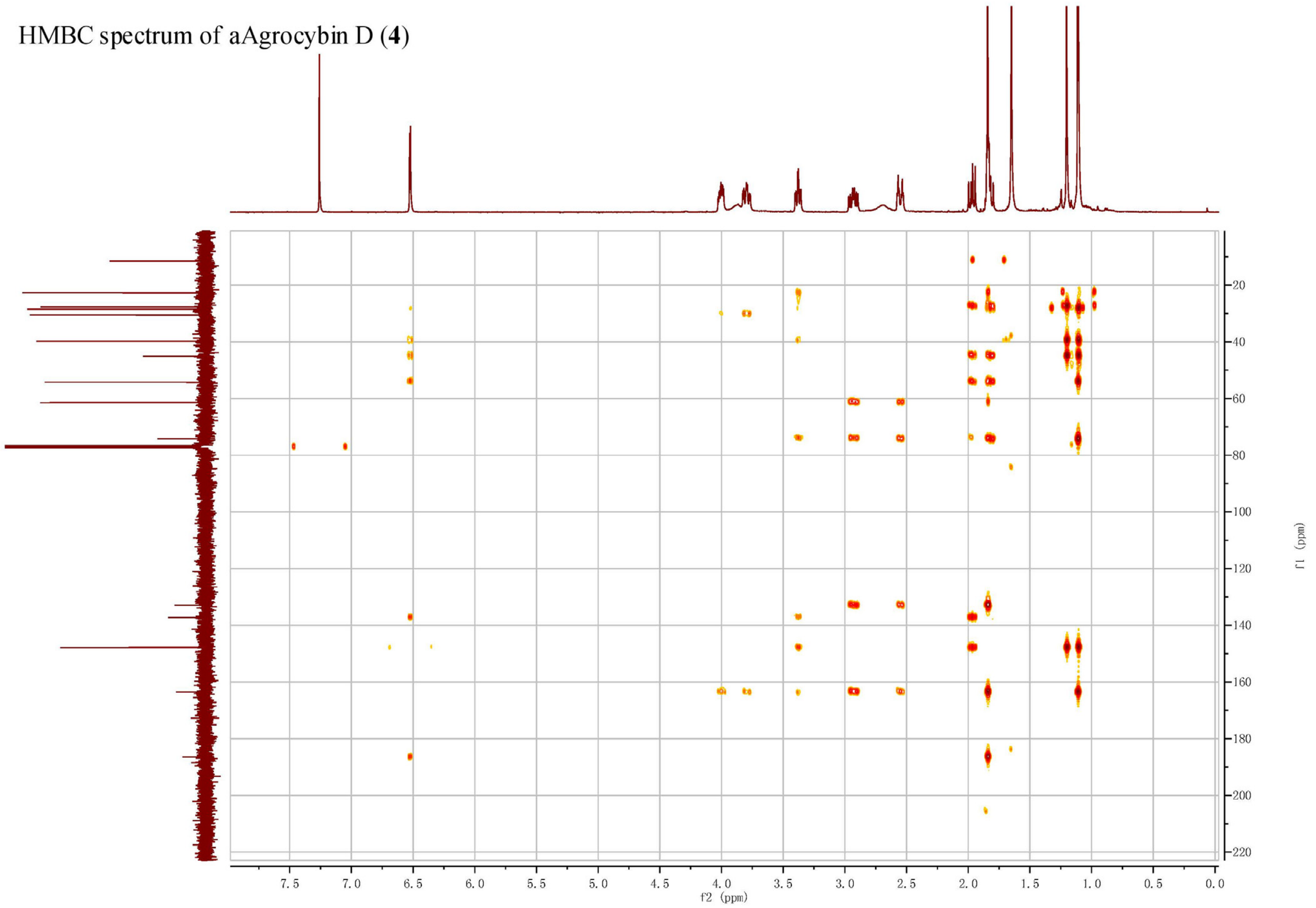

ROESY spectrum of agrocybin D (**4**)

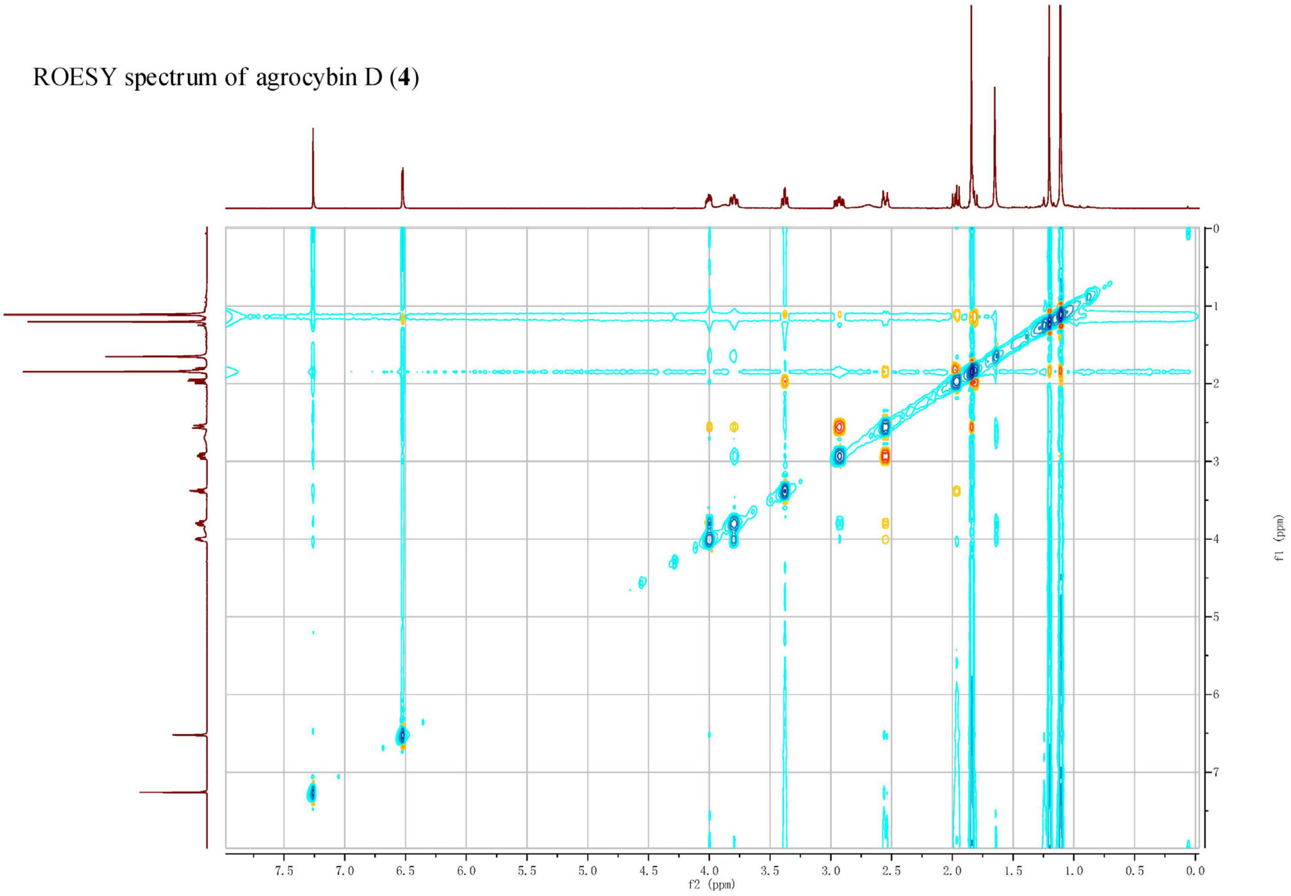

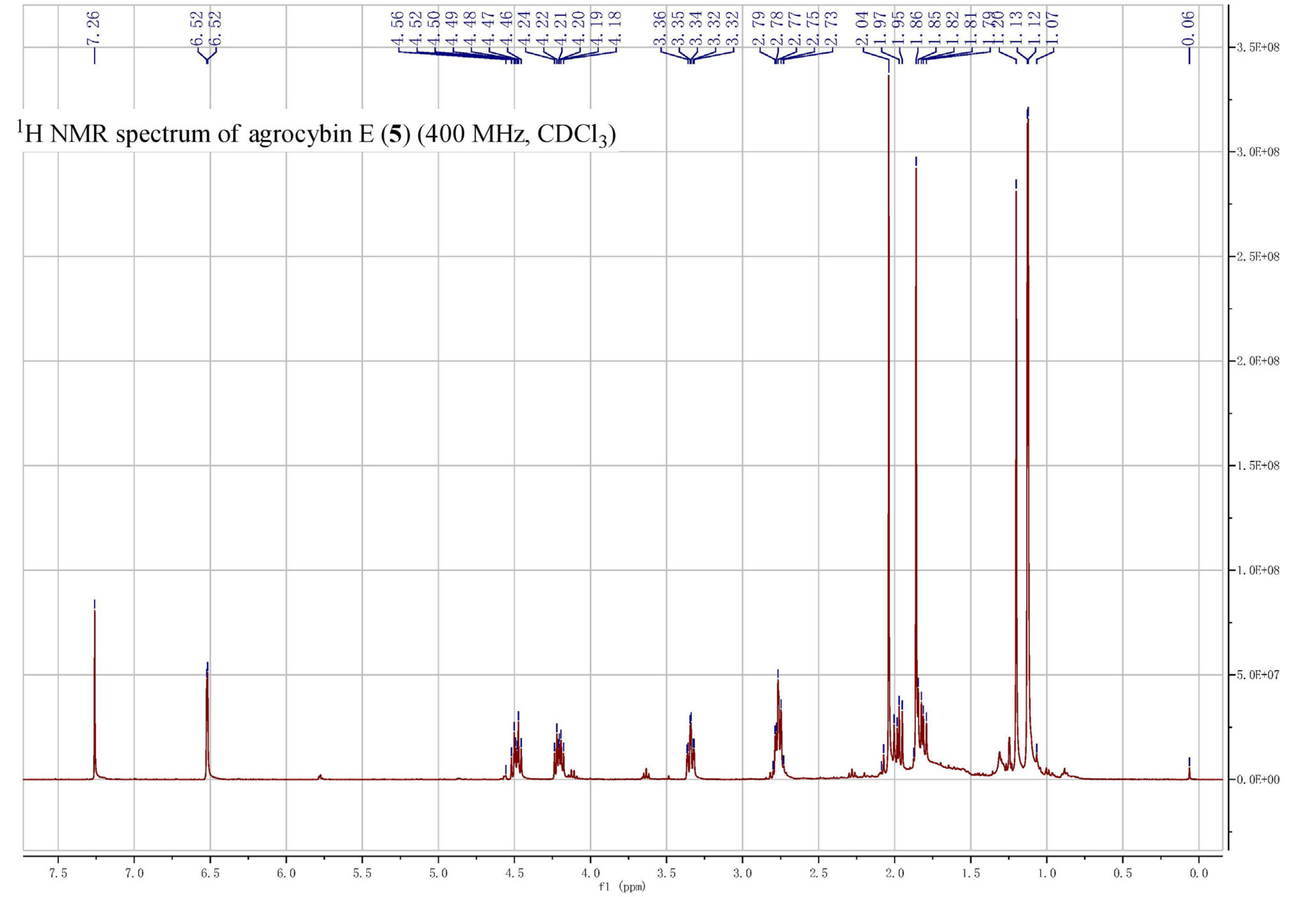
$^{1}$H NMR spectrum of agrocybin E (**5**) (400 MHz, $CDCl_3$)
f1 (ppm)

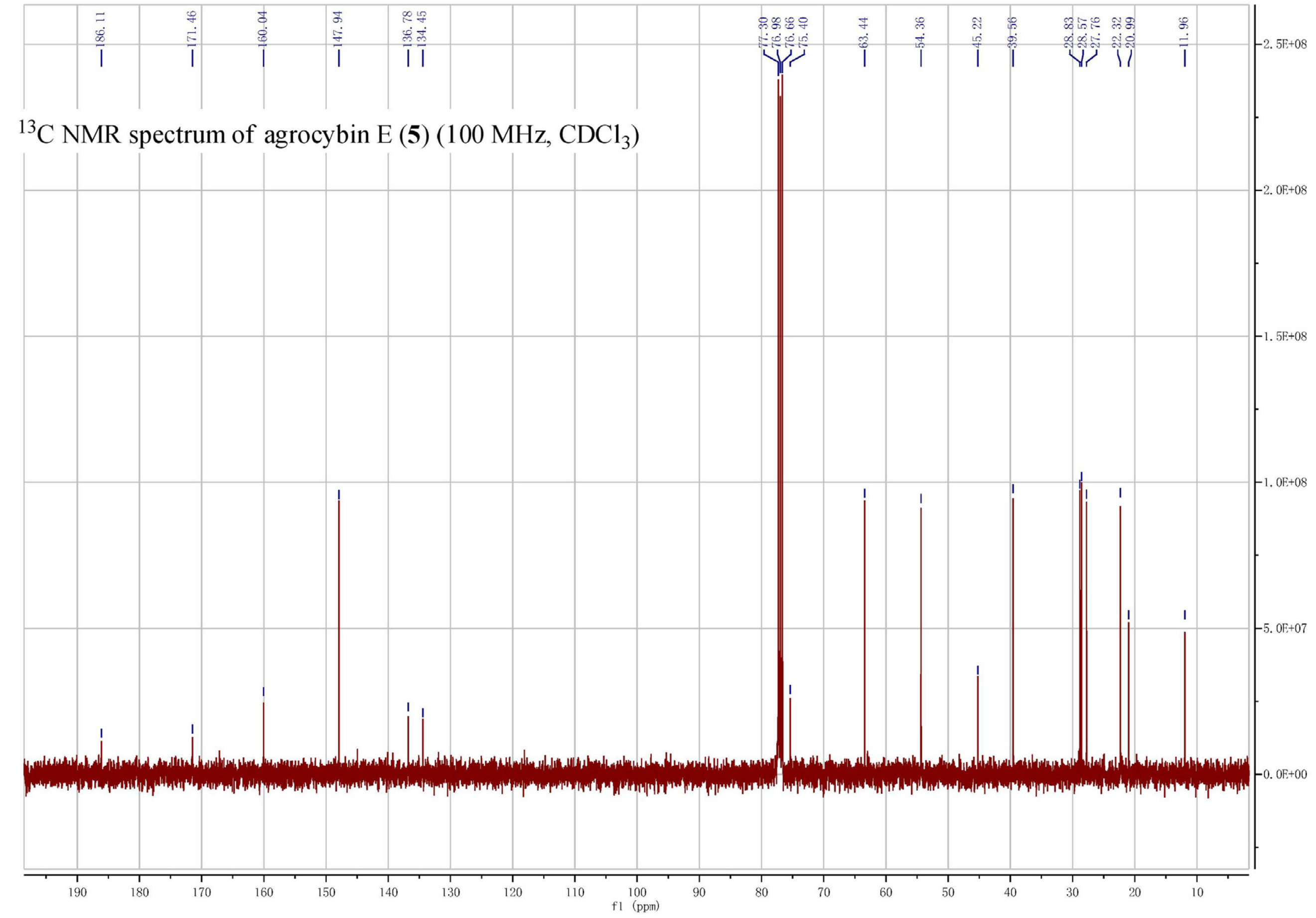
$^{13}$C NMR spectrum of agrocybin E (**5**) (100 MHz, $CDCl_3$)
f1 (ppm)

HMBC spectrum of agrocybin E (**5**)

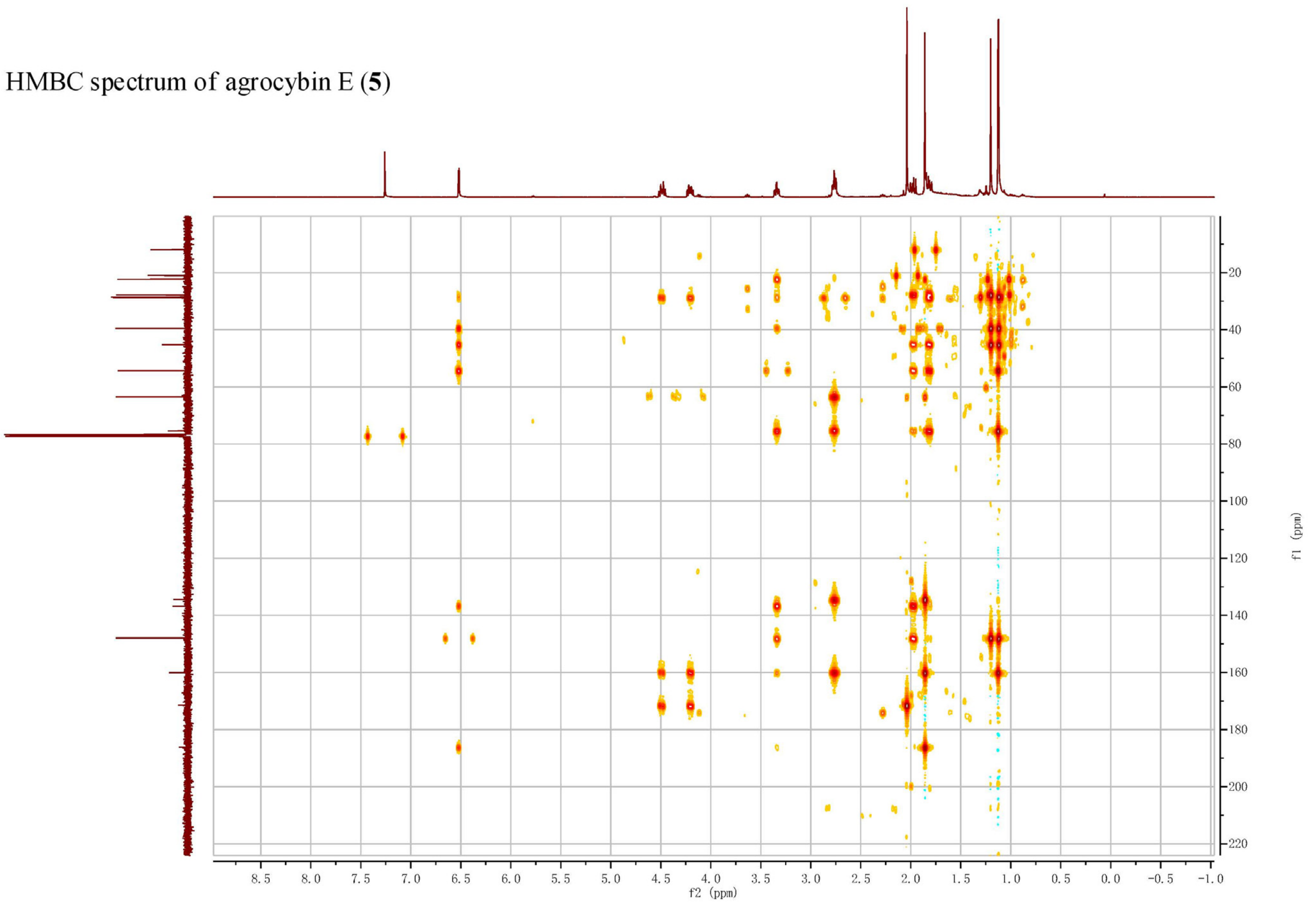

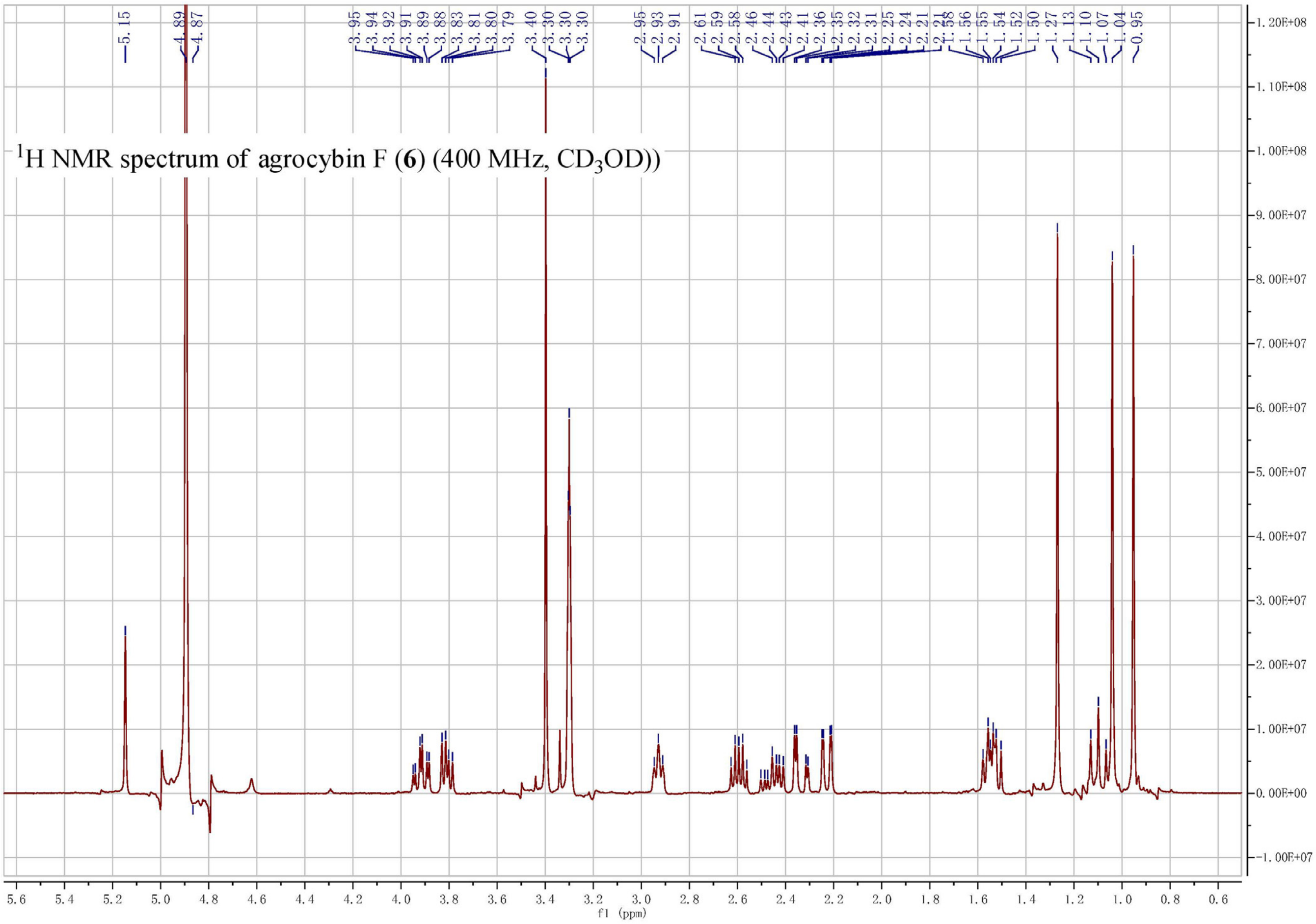
$^1$H NMR spectrum of agrocybin F (**6**) (400 MHz, $CD_3OD$))
f1 (ppm)

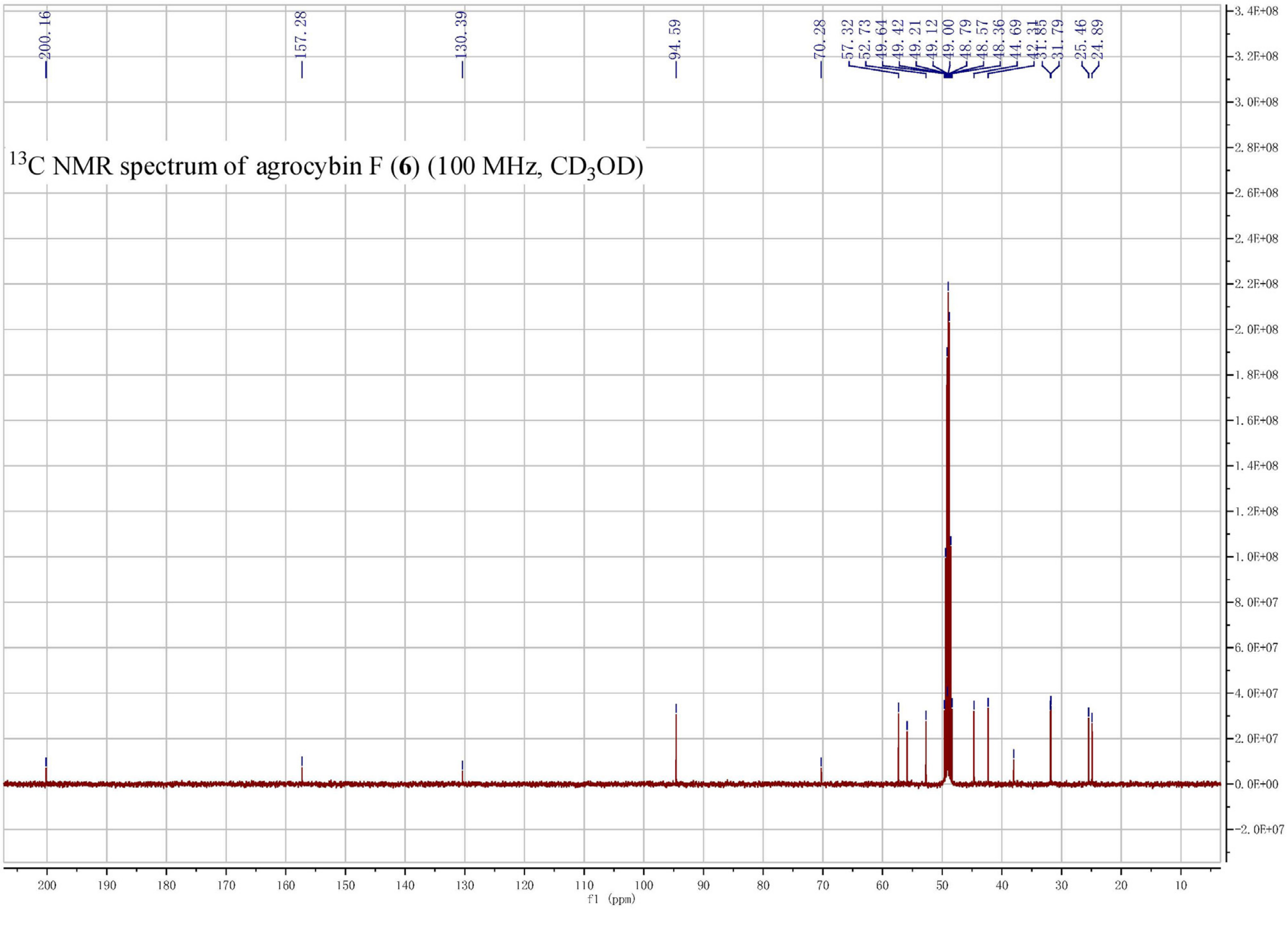
$^{13}$C NMR spectrum of agrocybin F (**6**) (100 MHz, $CD_3OD$)
f1 (ppm)

HMBC spectrum of agrocybin F (**6**)

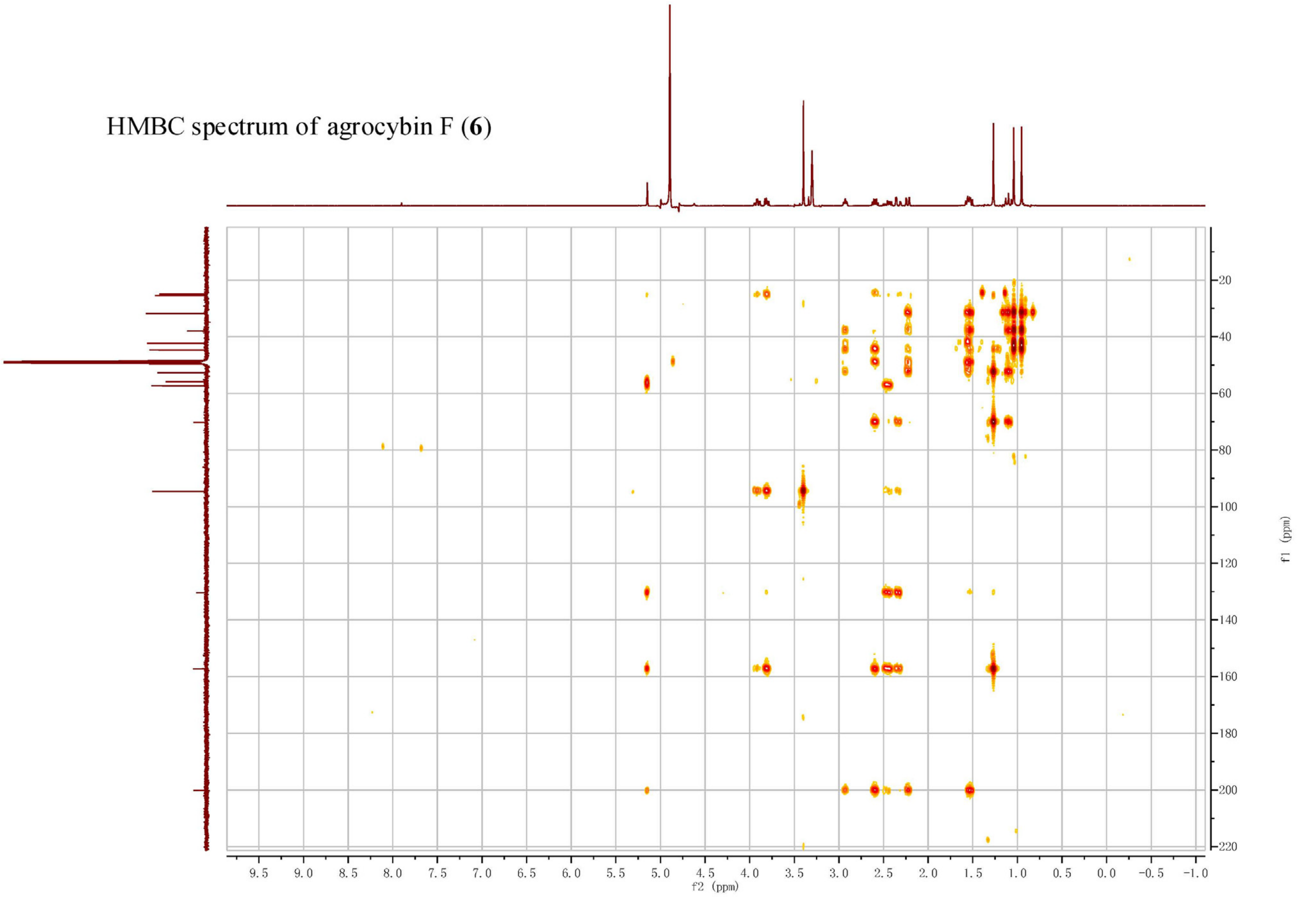


HSQC spectrum of agrocybin F (**6**)

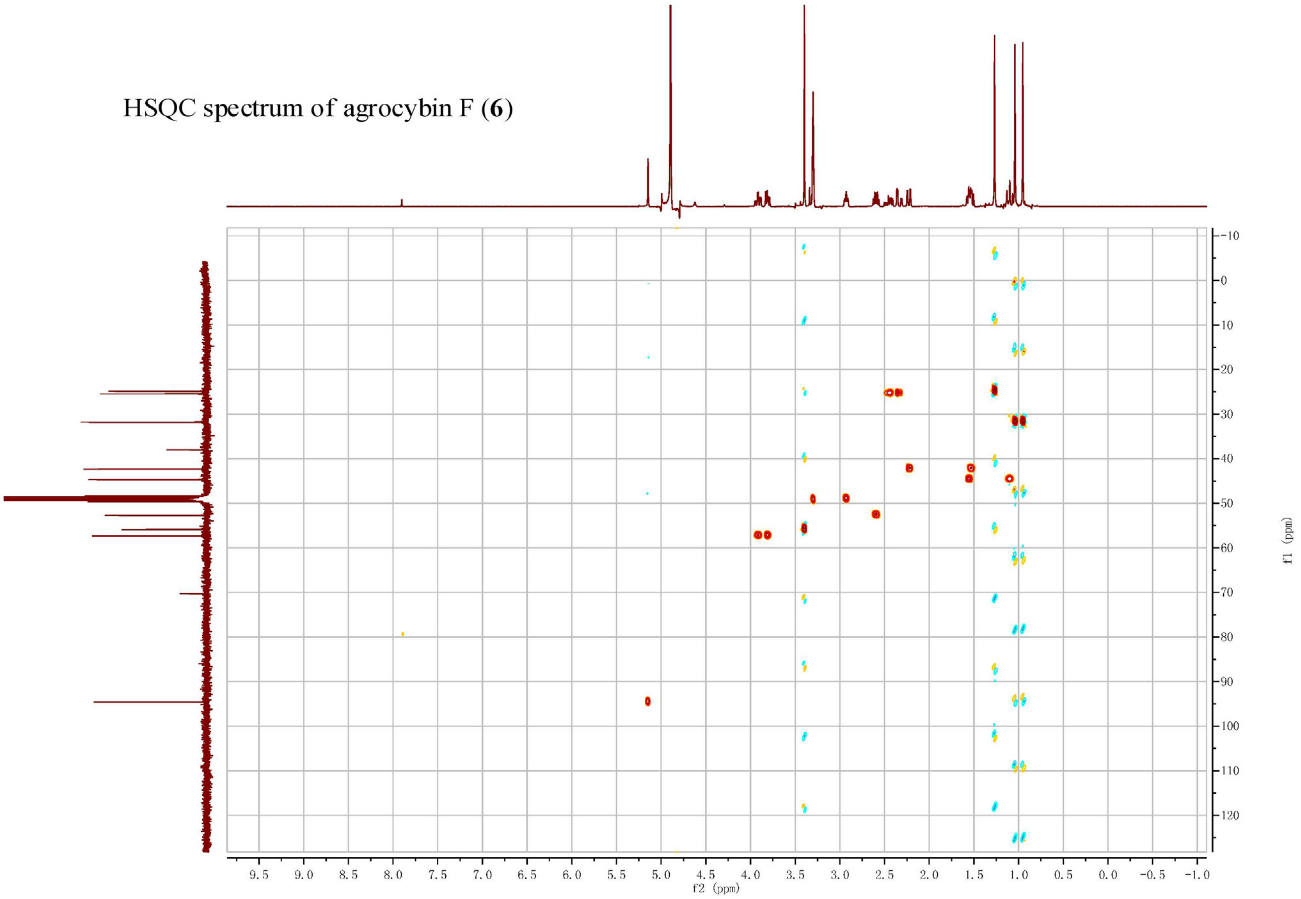

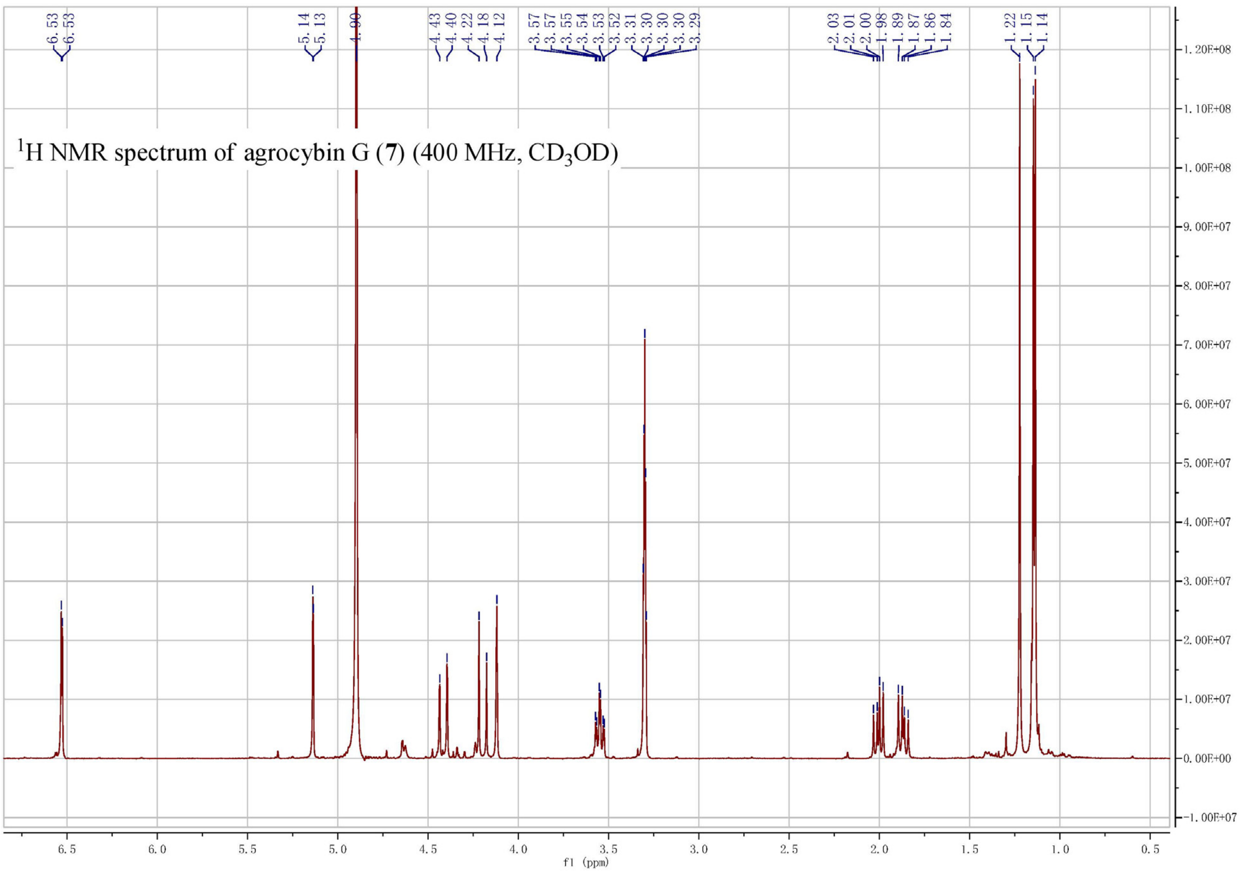

1H NMR spectrum of agrocybin G (7) (400 MHz, CD3OD)
6.53
6.53
5.14
5.13
4.90
4.43
4.40
4.22
4.18
4.12
3.57
3.57
3.55
3.54
3.53
3.52
3.31
3.30
3.30
3.30
3.29
2.03
2.01
2.00
1.98
1.89
1.87
1.86
1.84
1.22
1.15
1.14
f1 (ppm)


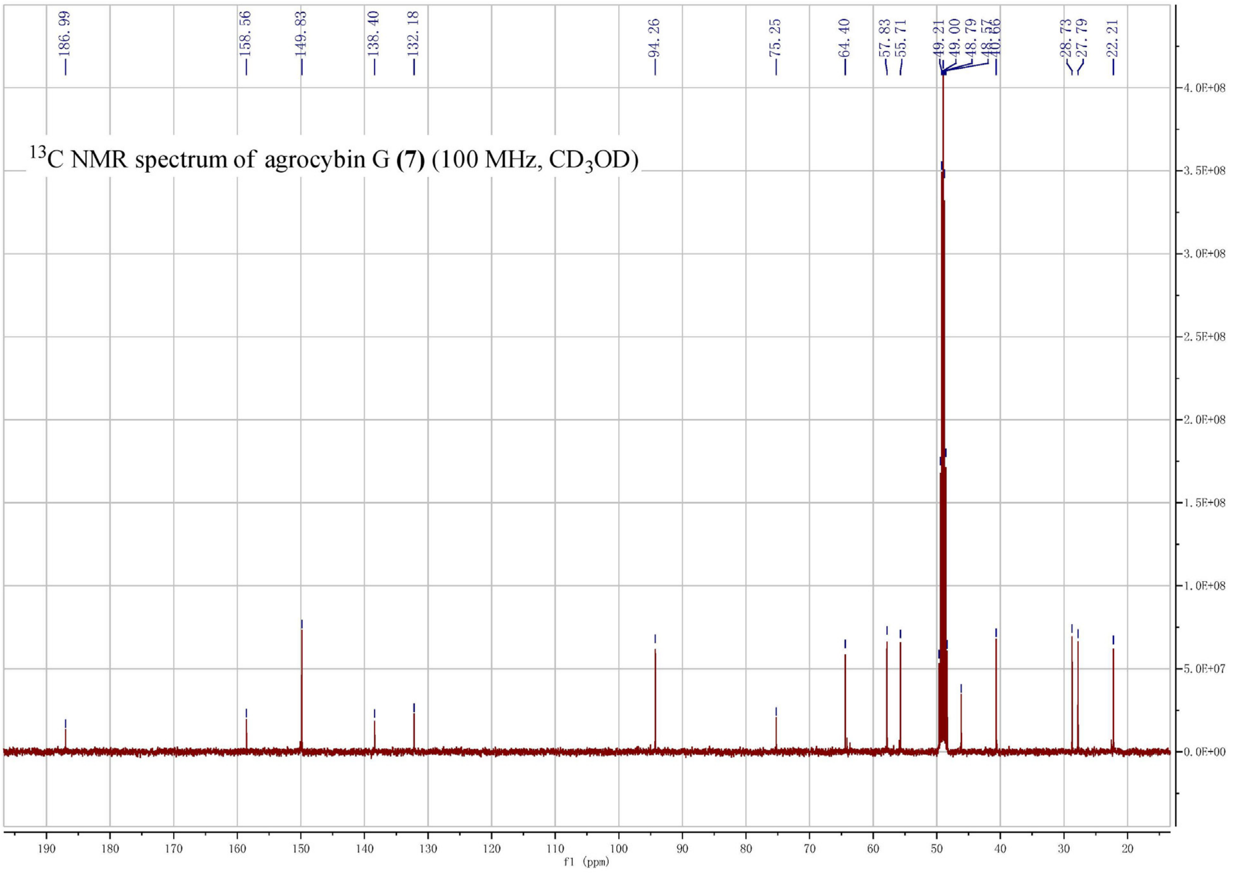

13C NMR spectrum of agrocybin G (7) (100 MHz, CD3OD)
186.99
158.56
149.83
138.40
132.18
94.26
75.25
64.40
57.83
55.71
49.21
49.00
48.79
48.57
40.66
28.73
27.79
22.21
f1 (ppm)

COSY spectrum of agrocybin G (**7**)

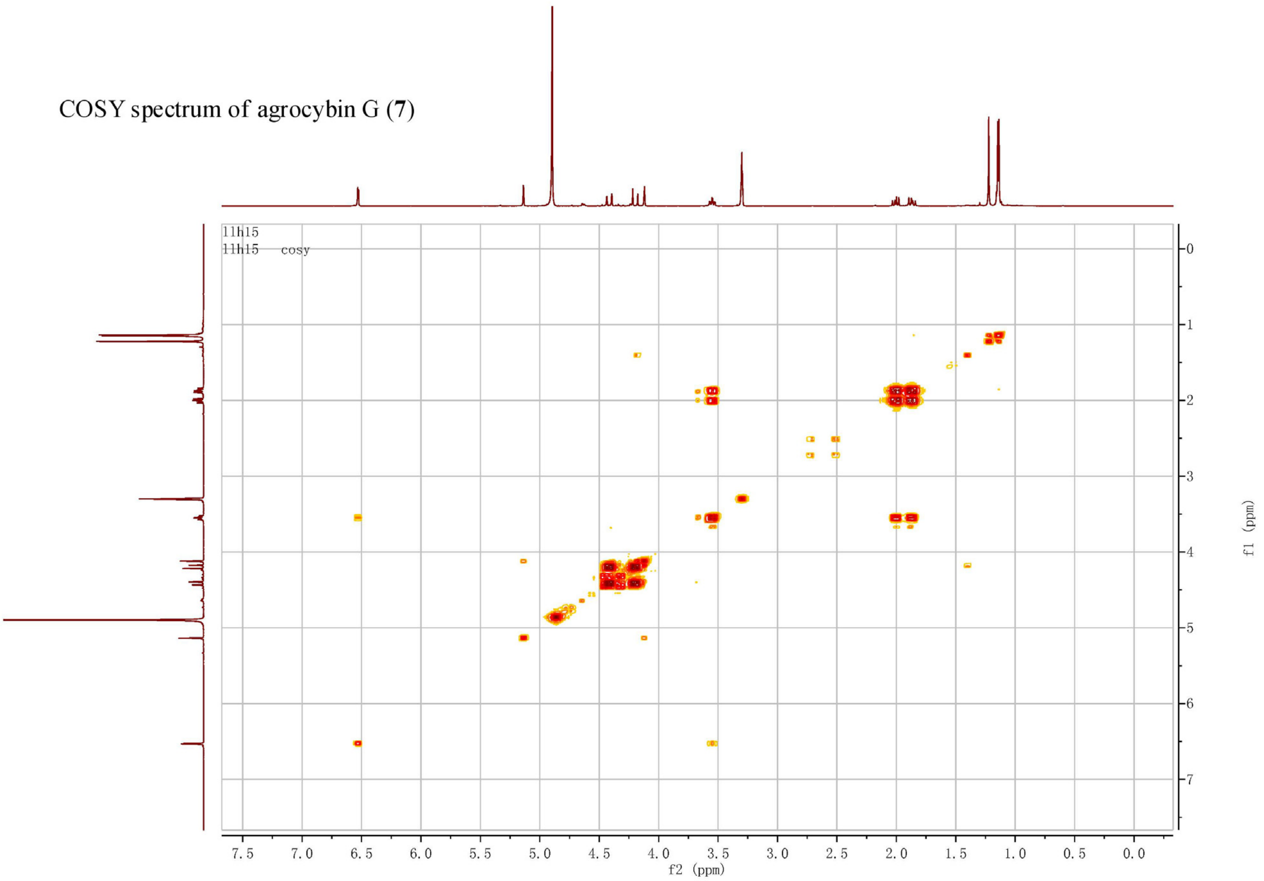


HSQC spectrum of agrocybin G (**7**)

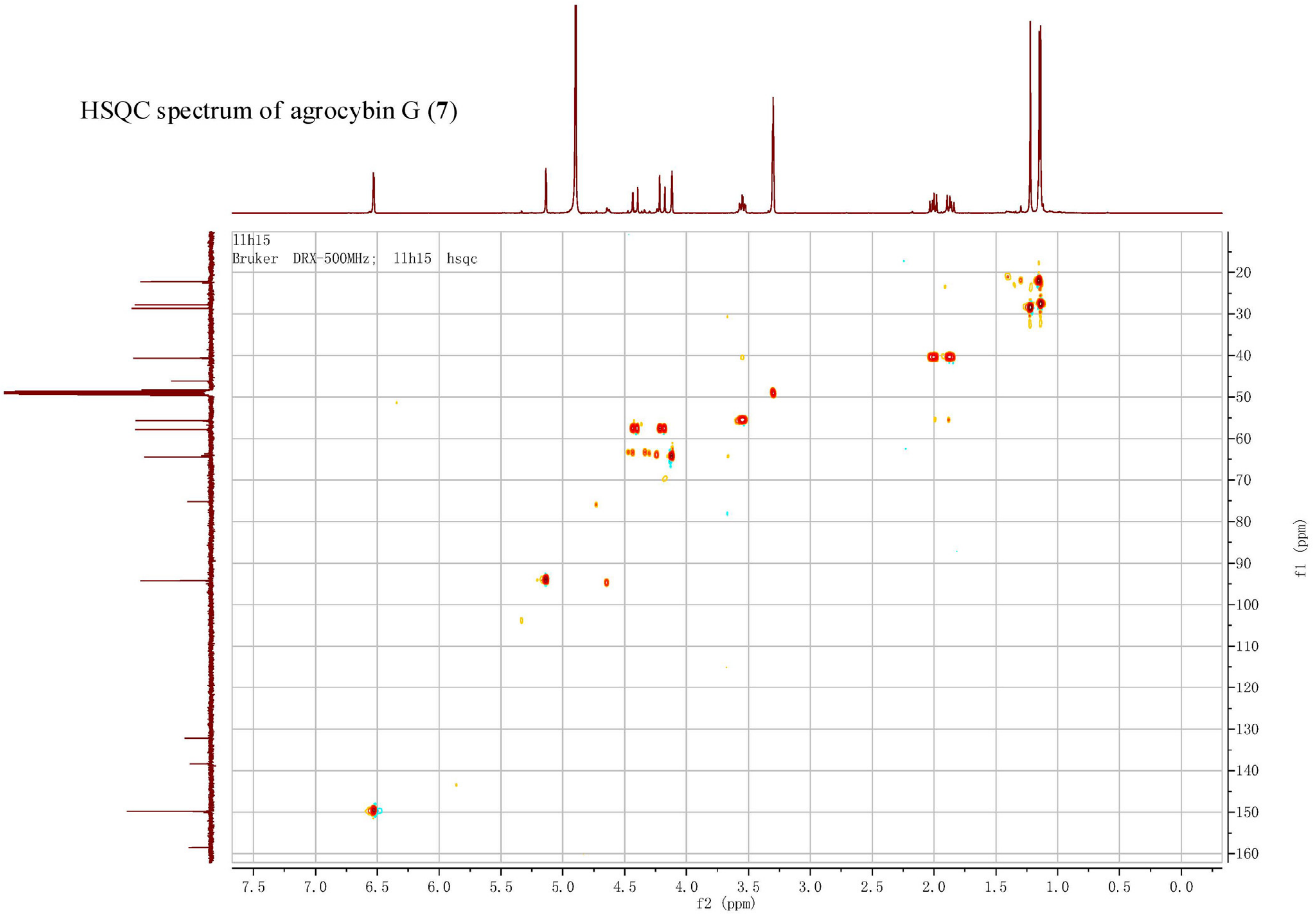

HMBC spectrum of agrocybin G (**7**)

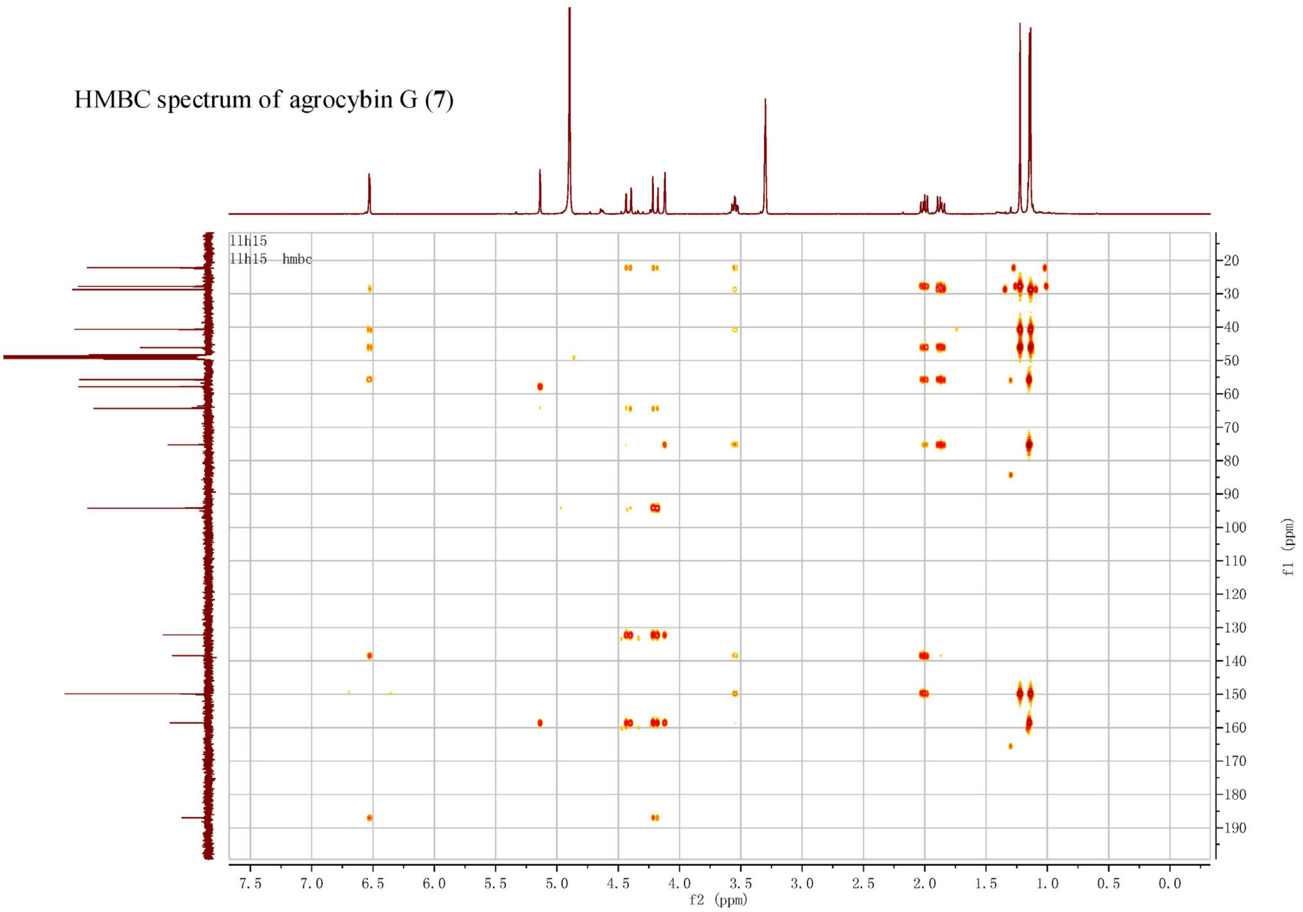


ROESY spectrum of agrocybin G (**7**)

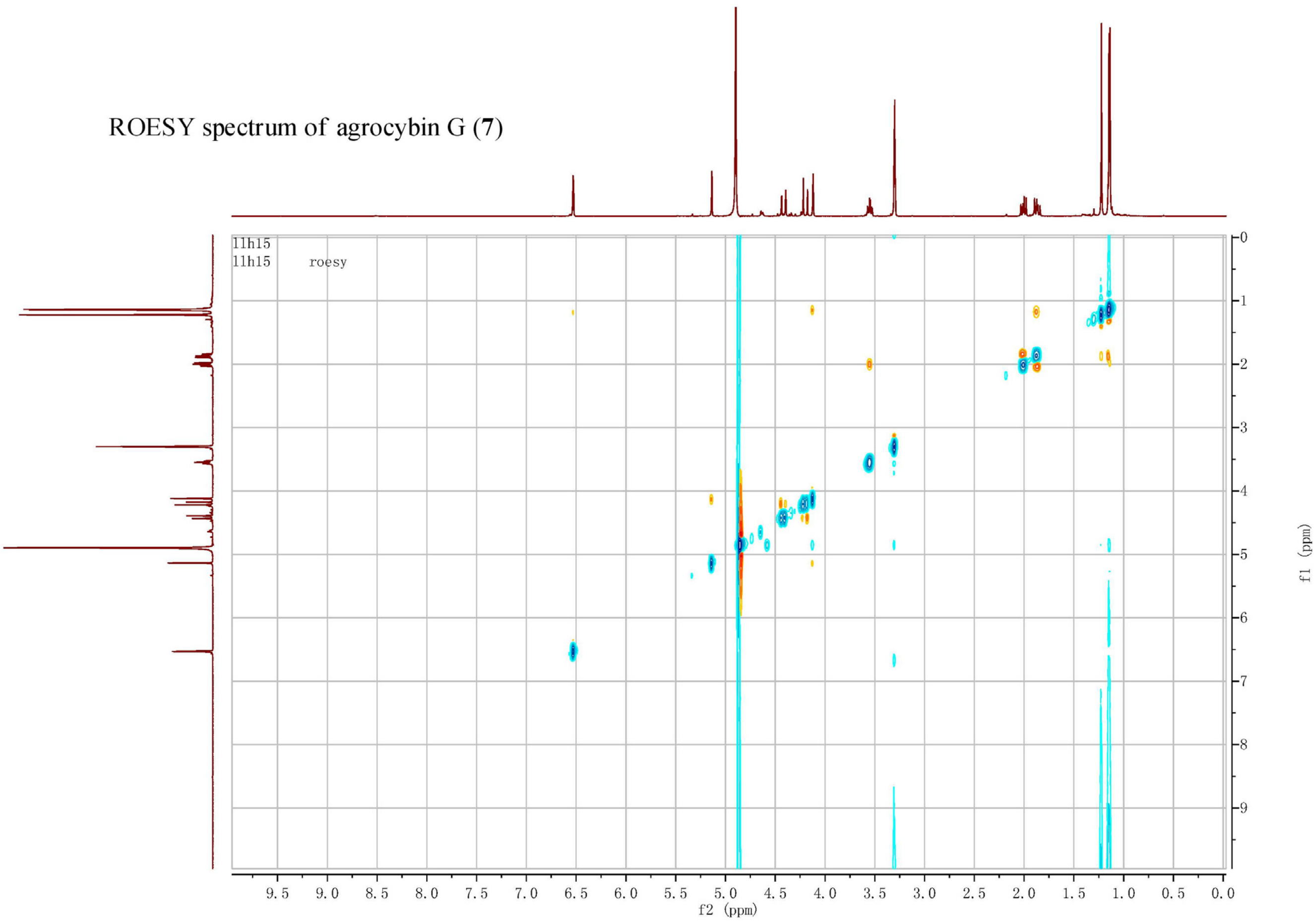

Supplement: Supplementary file 1 — Supplementary material, approximately 11.2 MB. [file 13659_2011_18_MOESM1_ESM.pdf]
